# Periodontitis Induces B Cell‐Macrophage Crosstalk to Exacerbate Glucose Dysregulation in Obesity

**DOI:** 10.1002/advs.202517653

**Published:** 2026-03-20

**Authors:** Wen‐Zhen Lin, Lu‐Jun Zhou, Hui‐Lin Ye, Ting Liu, Bo‐Yan Chen, Xue‐Bing Bai, Jun Zhang, Lan Bai, Lin‐Juan Du, Yuan Liu, Yong‐Li Wang, Hong Zhu, Yu‐Lin Li, Shuo Xu, Xiao‐Qian Meng, Guo‐Cai Tian, Yan Liu, Wu‐Chang Zhang, Ya‐Qin Zhu, Sheng‐Zhong Duan

**Affiliations:** ^1^ Department of General Dentistry Shanghai Ninth People's Hospital Shanghai Jiao Tong University School of Medicine Shanghai China; ^2^ Laboratory of Oral Microbiota and Systemic Diseases, Shanghai Ninth People's Hospital College of Stomatology, Shanghai Jiao Tong University National Center for Stomatology National Clinical Research Center for Oral Diseases Shanghai Key Laboratory of Stomatology Shanghai Research Institute of Stomatology Shanghai China; ^3^ Stomatology Hospital, School of Stomatology, and Institute of Translational Medicine Zhejiang University School of Medicine Zhejiang Provincial Clinical Research Center for Oral Diseases Key Laboratory of Oral Biomedical Research of Zhejiang Province Engineering Research Center of Oral Biomaterials and Devices of Zhejiang Province Hangzhou China; ^4^ State Key Laboratory of Transvascular Implantation Devices Hangzhou China

**Keywords:** B cell, macrophage, NLRP3/IL‐18 axis, periodontitis, type 2 diabetes

## Abstract

A growing consensus indicates that periodontitis (PD) adversely affects glycemic control in type 2 diabetes, though its underlying mechanisms remain unclear. Here, we report that PD, induced by long‐term oral ligatures, significantly aggravated hyperglycemia and hepatic gluconeogenesis in high‐fat diet‐induced obese mice, with its impact on insulin resistance being more pronounced in the liver than in adipose or muscle tissue. Immunologically, PD increased systemic B cell abundance, promoted hepatic B2 cell mobilization, facilitated the expansion of Kupffer cells and adipose tissue macrophages, and activated the NLRP3 inflammasome. Depleting B cells significantly alleviated PD‐induced glucose dysregulation, whereas adoptive transfer of B cells from PD mice exacerbated glucose dysregulation and Kupffer cell expansion in *Rag1^–/–^* mice. Mechanistically, upregulation of the interleukin (IL)‐18 receptor was observed in PD‐exposed B cells. IL‐18 directly enhanced B cell proliferation, and hepatic macrophages from PD mice secreted elevated levels of IL‐18, further driving B cell expansion. Neutralizing IL‐18 or depleting macrophages significantly mitigated PD‐associated metabolic and B cell abnormalities. Therefore, PD exacerbates hyperglycemia by promoting the pathogenic expansion of B cells and their crosstalk with macrophages via the IL‐18 signaling axis. Targeting the NLRP3/IL‐18 axis holds promise for preventing glucose dysregulation and aberrant immune cell interactions primed by PD.

## Graphical abstract

In obesity, periodontitis exacerbates hyperglycemia and exhibits a liver‐centric preference for glucose regulation. This occurs through a periodontitis‐driven circuit between pathogenic B cells and hepatic macrophages. Their reciprocal expansion, orchestrated by the NLRP3/IL‐18 signaling pathway, amplifies metabolic inflammation and disrupts glucose homeostasis, unveiling a targetable axis linking oral infection to systemic metabolism.

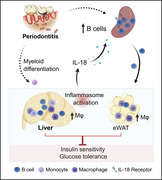

## 1 Introduction

Obesity causes fat deposition in the liver and adipose tissue, leading to a constellation of metabolic abnormalities and diseases [1]. Type 2 diabetes (T2D), a heterogeneous disease with the hallmarks of β‐cell dysfunction and insulin resistance [2], is increasingly prevalent owing to sedentary lifestyles and obesogenic environments [3]. Estimates indicate that by 2045, the global prevalence of T2D among individuals aged 20–79 will rise to 12.2%, imposing a substantial socioeconomic and clinical burden [4]. Although a wide array of antidiabetic medications is available, their effectiveness remains unsatisfactory because of poor glycemic control or long‐term complications [5]. Therefore, elucidating the underlying mechanisms of newly recognized risk factors is essential for developing novel strategies to combat the T2D epidemic.

The concept of oral health as a critical determinant of overall wellness has been proposed for years [6]. Homeostatic defects in the oral ecosystem may compromise the host's responses to various pathologies. As a paradigm of dysbiosis‐driven oral disease, periodontitis (PD) not only devastates the supporting structures of teeth [7], but also predisposes individuals to systemic vulnerabilities [8, 9]. Recent epidemiological and mechanistic studies have connected PD with a range of diseases, including cardiovascular diseases, autoimmune diseases, and metabolic disorders [10, 11]. Intriguingly, oral pathogens and their products can inappropriately access remote sites through the ulcerated periodontal pockets [12] or along the alimentary tract [13, 14], thereby perturbing homeostasis both locally and systemically [15]. In the context of obesity, however, the target tissue most prominently affected by PD has yet to be identified.

It has been unequivocally established that PD and T2D are highly interrelated; PD adversely affects glycemic control in patients with T2D, and T2D accelerates the destruction of periodontal supporting tissues [16]. Immune dysfunction and chronic inflammation are shared pathological features that fuel the progression of both T2D and PD [17]. Pro‐inflammatory effector T cells, M1‐like macrophages, and antibody‐producing B2 cells increase in T2D, thus promoting metabolic disturbances [3]. Meanwhile, PD drives myeloid differentiation with enhanced pro‐inflammatory potential, which is linked to inflammatory comorbidities [18]. However, the extent to which immune changes elicited by PD influence T2D pathogenesis remains elusive. Analyses reflecting the phenotypic and functional attributes of immune cells are required to capture the systemic immunological imprint of PD on T2D.

In the present study, we aimed to delineate the metabolic and immune effects of PD on T2D and to identify the underlying mechanistic causality. We first comprehensively characterized the PD‐associated metabolic and immune phenotypes across insulin‐sensitive organs in high‐fat diet (HFD)‐induced obese mice with long‐term ligature‐induced PD. Next, we identified the targetable immune cells through which PD predisposed the host to glucose disorders via cell depletion and adoptive transfer approaches. Furthermore, we investigated the molecular mechanisms underlying the pathogenic immune cell expansion triggered by PD using RNA sequencing, cell depletion, cytokine neutralization, and co‐culture experiments.

## 2 Results

### 2.1 PD Exacerbates HFD‐Induced Glucose Dysregulation

**FIGURE 1:**
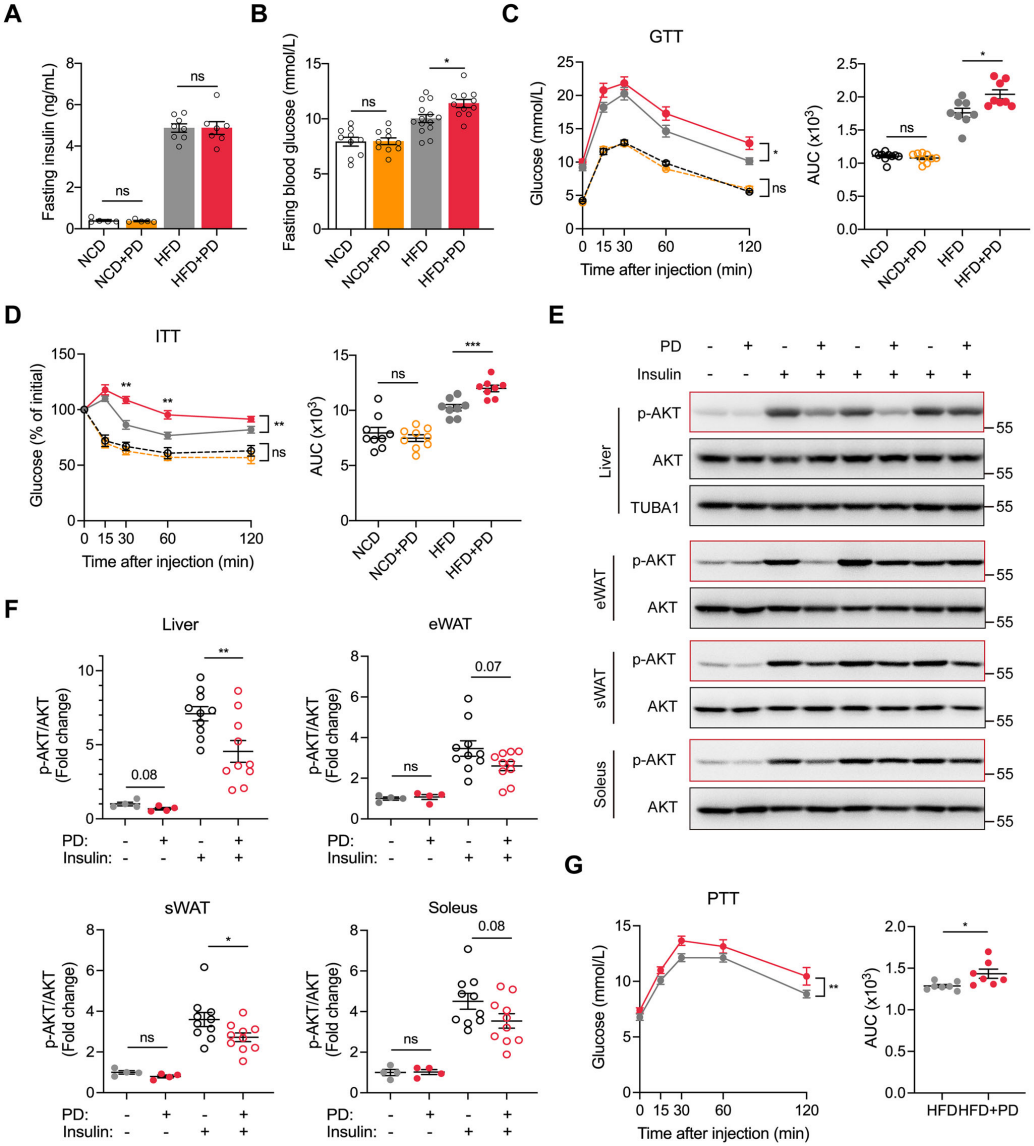
Periodontitis (PD) exacerbates insulin resistance and glucose intolerance in obese mice. (A) Serum insulin levels after a 5 h fast. (B) Blood glucose levels after a 5 h fast. (C) Glucose tolerance test (GTT) and area under the curve (AUC) analysis. (D) Insulin tolerance test (ITT) with AUC quantification. (E) Representative immunoblots showing phosphorylated AKT (p‐AKT) and total AKT in the liver, epididymal white adipose tissue (eWAT), subcutaneous white adipose tissue (sWAT), and soleus muscle following intraperitoneal injection of saline (–) or insulin (+). (F) Densitometric quantification of the immunoblots exemplified in (E). (G) Pyruvate tolerance test (PTT) with AUC quantification. Data are shown as mean ± SEM. Each data point represents an individual mouse. Unpaired Student's *t*‐test (A, B, F; right parts of C, D, and G) and two‐way ANOVA (left parts of C, D, and G) were used for statistical analysis. ns, not significant. ^*^ *p* < 0.05, ^**^ *p* < 0.01, ^***^ *p* < 0.001.

A ligature‐induced PD model was established to recapitulate human periodontal disease and to investigate the effects of oral inflammation on T2D progression in HFD‐induced obese mice. Consistent with previous studies [19, 20], PD caused apparent alveolar bone resorption in both normal chow diet (NCD)‐fed and HFD‐fed mice (Figure S1A–C), as characterized by loss of connective tissue attachment and disruption of the gingival epithelium (Figure S1D). HFD feeding induced the expected metabolic derangements, such as obesity, hyperglycemia, glucose intolerance, and insulin resistance, regardless of PD status, confirming successful establishment of obesity (Figure S2A; Figure 1A–D). Furthermore, PD exacerbated multiple facets of metabolic dysfunction in obese mice, including significantly elevated fasting blood glucose (Figure 1B), worsened glucose tolerance (Figure 1C), and increased insulin resistance (Figure 1D). Concurrently, PD suppressed insulin‐dependent AKT phosphorylation in the hepatic, adipose, and muscle tissues, suggesting insulin signaling impairment in obese mice (Figure 1E,F). Specifically, PD had a more pronounced impact on hepatic insulin resistance than on adipose tissue or soleus muscle, highlighting a preferential oral–liver axis. Therefore, we investigated hepatic gluconeogenesis, a process that plays a crucial role in systemic glucose homeostasis [21]. A pyruvate tolerance test (PTT) demonstrated that PD significantly increased hepatic glucose output in obese mice (Figure 1G). In contrast, PD did not alter body weight, food intake, or liver and adipose tissue weights (Figure S2), nor did it influence serum or hepatic lipid levels in this composite model (Figure S3A–E). Taken together, PD specifically aggravated obesity‐related glucose metabolic disturbances, particularly hepatic insulin resistance and gluconeogenesis, without affecting adiposity or lipid profiles.

### 2.2 PD Promotes Hepatic Injury and Alters the Hepatic Immune Microenvironment in Obese Mice

**FIGURE 2:**
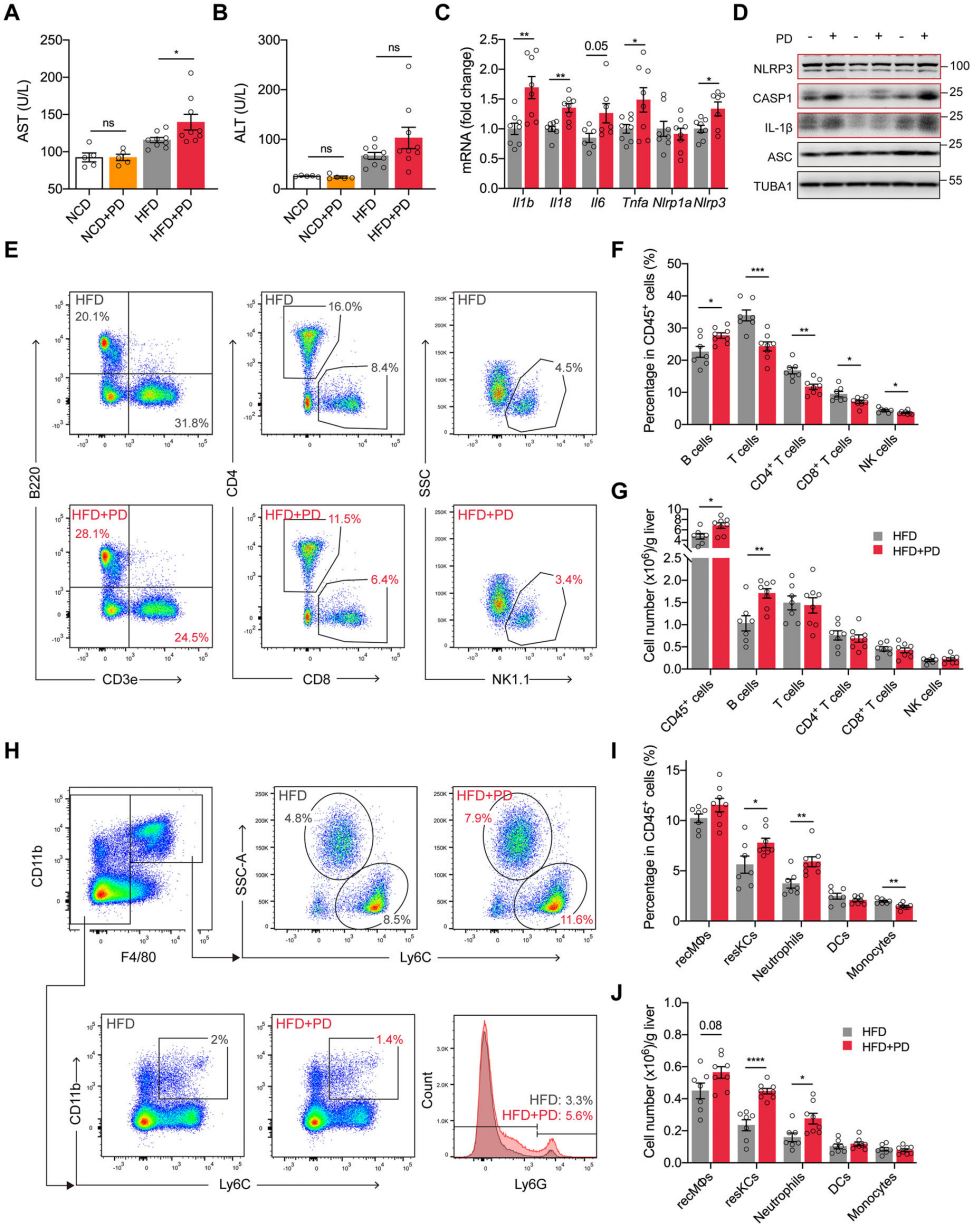
PD exacerbates hepatic injury and remodels the immune microenvironment in obese mice. (A) Serum aspartate aminotransferase (AST) levels. (B) Serum alanine aminotransferase (ALT) levels. (C) Quantitative real‐time PCR (qRT‐PCR) analysis of inflammatory cytokines and inflammasome‐related genes in the liver. (D) Immunoblots of NLRP3 inflammasome signaling components in the liver. (E) Representative flow cytometry plots showing the proportions of B (B220^+^CD3e^−^), T (B220^−^CD3e^+^), CD4^+^ T, CD8^+^ T, and natural killer (NK) cells (B220^−^CD3e^−^NK1.1^+^) in the livers of obese mice. (F) Quantification of B, T, CD4^+^ T, CD8^+^ T, and NK cells as percentages of hepatic CD45^+^ cells. (G) Quantification of the numbers of total CD45^+^ cells and the indicated hepatic lymphocyte populations. (H) Representative flow cytometry plots showing the proportions of recruited macrophages (recMφs, F4/80^+^CD11b^+^Ly6C^hi^), resident Kupffer cells (resKCs, F4/80^+^CD11b^+^Ly6C^lo^), monocytes (F4/80^−^CD11b^+^Ly6C^+^), and neutrophils (Ly6G^+^) in the livers of obese mice. (I) Quantification of recMφs, resKCs, neutrophils, dendritic cells (DCs), and monocytes as percentages of hepatic CD45^+^ cells. (J) Quantification of the numbers of the indicated hepatic myeloid cell populations. Data are shown as mean ± SEM. Each data point represents an individual mouse. Unpaired Student's *t*‐test was used for statistical analysis. ns, not significant. ^*^ *p* < 0.05, ^**^ *p* < 0.01, ^***^ *p* < 0.001, ^****^ *p* < 0.0001.

Given the liver's pivotal role in PD‐induced glucose dysregulation, we next examined hepatic function and immune responses in obese mice. HFD feeding significantly elevated serum aspartate aminotransferase (AST) and alanine aminotransferase (ALT) levels. PD further increased AST levels, whereas ALT levels showed a non‑significant trend toward elevation (Figure 2A,B). This hepatic injury was accompanied by upregulation of pro‐inflammatory genes and activation of the NLRP3 inflammasome, as evidenced by elevated protein levels of NLRP3, CASP1, and IL‐1β, but not ASC, in obese mice (Figure 2C,D; Figure S3F). Flow cytometric analysis revealed that PD significantly enhanced hepatic immune cell infiltration (Figure 2E–J), with gating strategies shown in Figures S4 and S5. Notably, B cells, which represented the second most abundant hepatic immune cell population after T cells in HFD mice, exhibited significantly increased percentages and numbers following PD, becoming the predominant immune subset (Figure 2E–G). In contrast, although the percentages of T (CD4^+^ and CD8^+^) and natural killer (NK) cells decreased, their numbers remained unchanged (Figure 2E–G). We further characterized macrophage subsets, distinguishing resident Kupffer cells (resKCs) from recruited monocyte‐derived macrophages (recMφs) [22]. PD significantly expanded resKCs in both percentages and numbers, whereas recMφs exhibited a modest, non‐significant trend toward increase (Figure 2H–J). Simultaneously, neutrophil numbers were significantly increased in HFD+PD mice (Figure 2H–J). Although the percentages of monocytes declined, their numbers remained unchanged (Figure 2H–J). These results demonstrated that PD induced hepatic injury and promoted the recruitment of leukocytes, primarily B cells, along with the expansion of resKCs in obese mice.

### 2.3 PD Potentiates HFD‐Induced Adipose Tissue Inflammation

**FIGURE 3:**
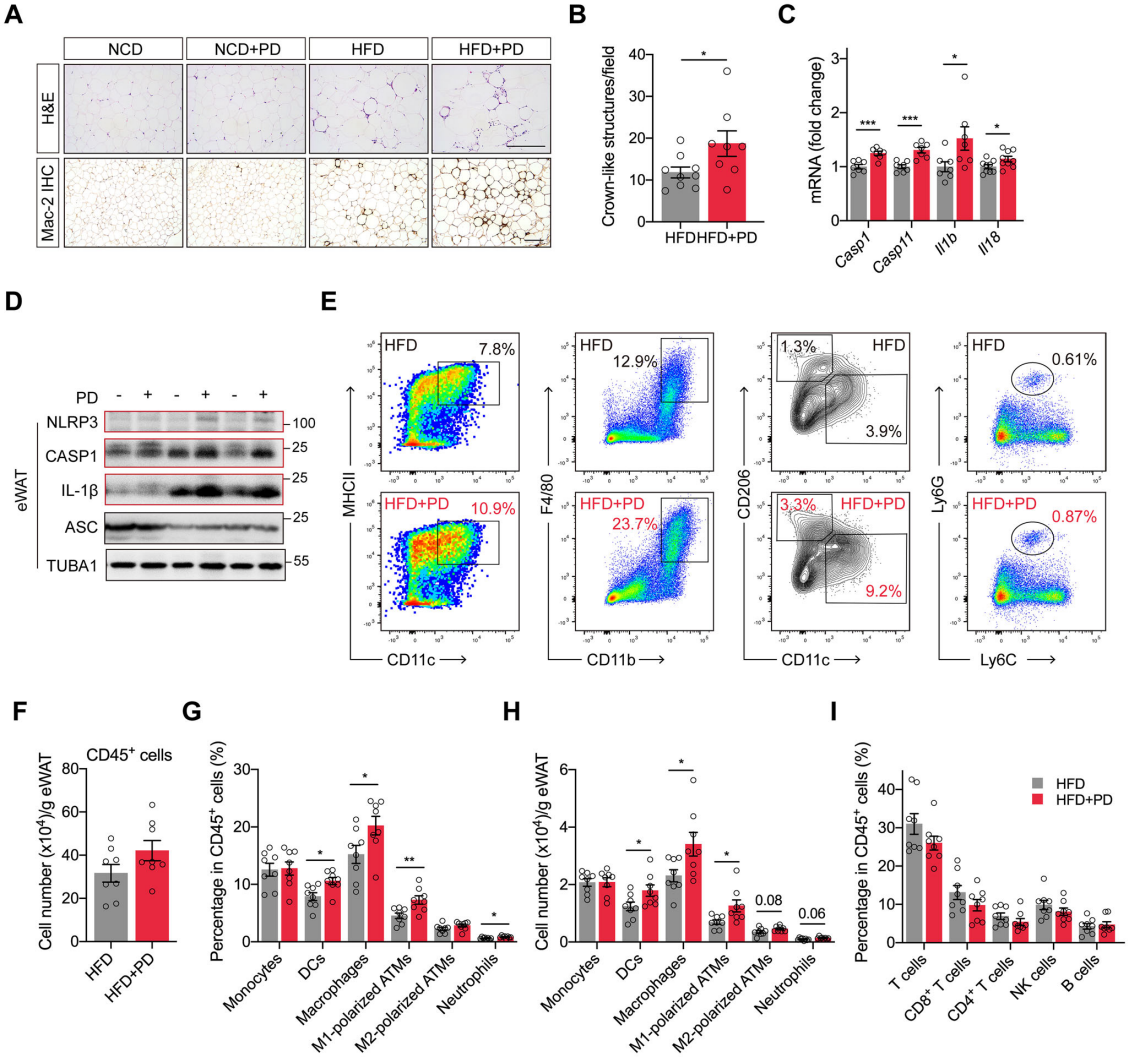
PD promotes inflammation and macrophage infiltration in the adipose tissue of obese mice. (A) Representative hematoxylin and eosin (H&E) staining and Mac‐2 immunohistochemistry (IHC) of eWAT sections. Scale bar, 200 µm. (B) Quantification of crown‐like structures in Mac‐2 IHC‐stained sections. (C−D) qRT‐PCR analysis (C) and immunoblots (D) of inflammasome‐related components in the eWAT of obese mice with (+) or without (–) PD. (E) Representative flow cytometry plots showing the proportions of DCs (CD11c^+^MHCII^+^), macrophages (CD11b^+^F4/80^+^), M1‐polarized adipose tissue macrophages (ATMs) (CD11c^+^CD206^−^), M2‐polarized ATMs (CD206^+^CD11c^−^), and neutrophils (Ly6G^+^) in eWAT. (F) Quantification of CD45^+^ cell numbers normalized to tissue weight. (G) Quantification of the indicated myeloid cell populations as percentages of CD45^+^ cells in eWAT. (H) Quantification of the numbers of the indicated myeloid cell populations in eWAT. (I) Quantification of T, CD4^+^ T, CD8^+^ T, NK, and B cells as percentages of CD45^+^ cells in eWAT. Data are shown as mean ± SEM. Each data point represents an individual mouse. Unpaired Student's *t*‐test was used for statistical analysis. ^*^ *p* < 0.05, ^**^ *p* < 0.01, ^***^ *p* < 0.001.

Given that PD also impaired insulin signaling in adipose tissue, we examined adipose tissue alterations, as inflamed adipose tissue is a key etiological feature of obesity‑associated insulin resistance [23]. Hematoxylin and eosin (H&E) staining showed that HFD feeding enlarged adipocytes in both epididymal white adipose tissue (eWAT) and subcutaneous white adipose tissue (sWAT), whereas PD did not further increase the adipocyte size in either depot (Figure 3A; Figure S6A). Mac‑2 staining revealed that crown‑like structures, sites where macrophages clear dying adipocytes [24], were more abundant in the eWAT of HFD mice than in NCD‐fed mice, with a further increase in HFD+PD mice (Figure 3A,B). Elevated *Ccl2* expression in the sWAT of HFD+PD mice also indicated enhanced macrophage infiltration (Figure S6B). Analysis of NLRP3 inflammasome‐related genes showed that PD upregulated *Casp1* and *Il1b* expression in the eWAT of HFD+PD mice at both the mRNA and protein levels, indicating inflammasome activation (Figure 3C,D; Figure S6C). Flow cytometric analysis, with gating strategies outlined in Figure S6D, demonstrated that PD substantially increased the percentages and numbers of dendritic cells (DCs), macrophages, and neutrophils in the eWAT of HFD+PD mice, consistent with modestly enhanced immune cell infiltration (Figure 3E–H). Moreover, PD significantly increased the percentages and numbers of M1‐polarized pro‐inflammatory adipose tissue macrophages (ATMs) in HFD+PD mice (Figure 3G,H). In contrast, PD did not alter the percentages or numbers of monocytes, T cells, NK cells, or B cells in eWAT (Figure 3G–I; Figure S6E). These findings suggested that the predominant changes in ATMs likely contributed to the propagation of adipose inflammation and insulin resistance triggered by PD.

### 2.4 PD Facilitates B Cell Maturation While Skewing Differentiation Toward B2 Cells

**FIGURE 4:**
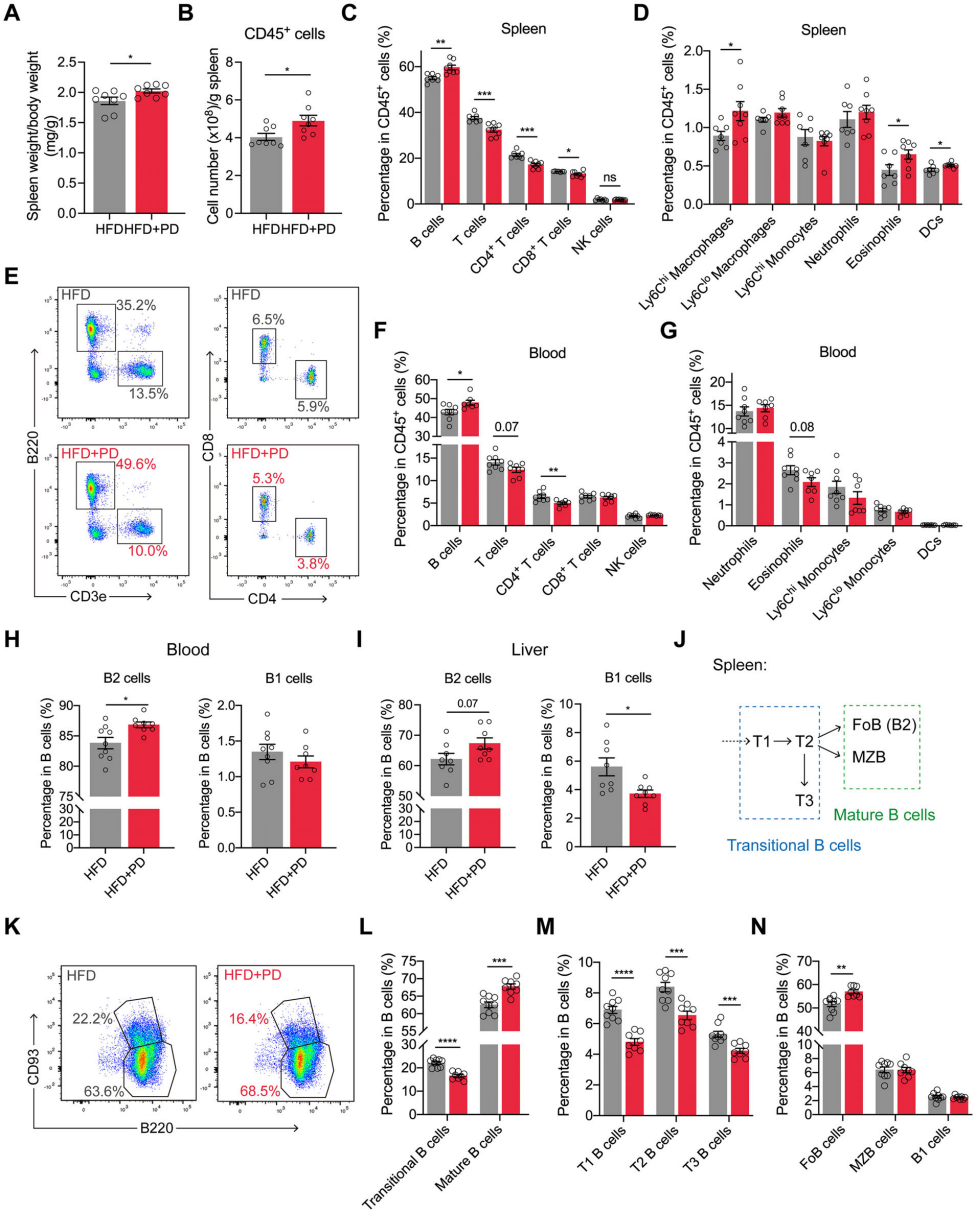
PD accelerates B cell maturation and differentiation into B2 cells in obese mice. (A) Ratio of spleen weight to body weight. (B) Quantification of splenic CD45^+^ cell numbers normalized to tissue weight. (C) Quantification of B, T (CD4^+^ and CD8^+^ T cells), and NK cells as percentages of CD45^+^ cells in the spleen. (D) Quantification of Ly6C^hi^ and Ly6C^lo^ macrophages, Ly6C^hi^ monocytes, neutrophils, eosinophils, and DCs as percentages of CD45^+^ cells in the spleen. (E) Representative flow cytometry plots showing the proportions of B and T cells (CD4^+^ and CD8^+^ T cells) in the blood of obese mice. (F) Quantification of the indicated lymphocyte populations as percentages of CD45^+^ cells in the blood. (G) Quantification of the indicated myeloid cell populations as percentages of CD45^+^ cells in the blood. (H−I) Quantification of B2 and B1 cells as percentages of total B cells in the blood (H) and the liver (I). (J) Schematic of peripheral B cell maturation stages in the spleen. (K) Representative flow cytometry plots showing the proportions of transitional (CD93^+^B220^+^) and mature B cells (CD93^−^B220^+^) in the spleen. (L−N) Quantification of transitional and mature B cells (L), transitional B cell subsets (M), and mature B cell subsets (N) as percentages of total B cells in the spleen. Data are shown as mean ± SEM. Each data point represents an individual mouse. Unpaired Student's *t*‐test was used for statistical analysis. ^*^ *p* < 0.05, ^**^ *p* < 0.01, ^***^ *p* < 0.001, ^****^ *p* < 0.0001.

To investigate the systemic immune responses of PD in obesity, we evaluated immune cell populations in the spleen, a key gatekeeper of systemic immunity [25], using standardized gating strategies (Figures S4 and S7) [26]. PD induced significant splenomegaly with elevated CD45^+^ cellularity (Figure 4A,B), which was characterized by a significant increase in the proportion of B cells with a concomitant decrease in T cells (Figure 4C), as well as an expansion of certain myeloid cell populations (Figure 4D). This B cell bias extended into the circulation (Figure 4E,F), suggesting B cell‐specific mobilization. Although certain splenic myeloid subsets, including Ly6C^hi^ macrophages, eosinophils, and DCs, were increased modestly but significantly, myeloid subsets in peripheral blood showed no significant changes (Figure 4D,G). Given the functional heterogeneity of B cells in metabolic regulation [27], we next performed a detailed subset analysis (Figure S8A) [28, 29]. B2 cells predominated and were significantly increased in the peripheral blood of HFD+PD mice (Figure 4H). Additionally, B2 cells also showed a marked increase in the liver (Figure 4I; Figure S8B−D). Although hepatic B1 cells exhibited reduced percentages, their numbers were comparable between groups, despite a selective increase in B1b cell numbers (Figure 4I; Figure S8B–D). We next investigated the B cell lineage, focusing on the final developmental stages in which immature B cells differentiate into mature B cells [30] (Figure 4J; Figure S9A) [28]. The splenic B cell compartment in HFD+PD mice exhibited distinct maturational shifts; transitional B cells (across all three stages) showed significantly reduced percentages with comparable numbers, whereas mature B cells expanded significantly in both percentages and numbers (Figure 4K–M; Figure S9B,C). This expansion was predominantly composed of follicular B cells (FoB, also referred to as B2 cells [31]), which were increased significantly in both percentages and numbers, whereas marginal zone B cells and B1 cells remained unchanged (Figure 4N; Figure S9D). Collectively, these findings indicated that PD drove the preferential expansion of mature B2 cells in obese mice, presumably originating from splenic precursor populations.

### 2.5 PD‐Exposed B Cells Contribute to Glucose Dysregulation and resKC Expansion

**FIGURE 5:**
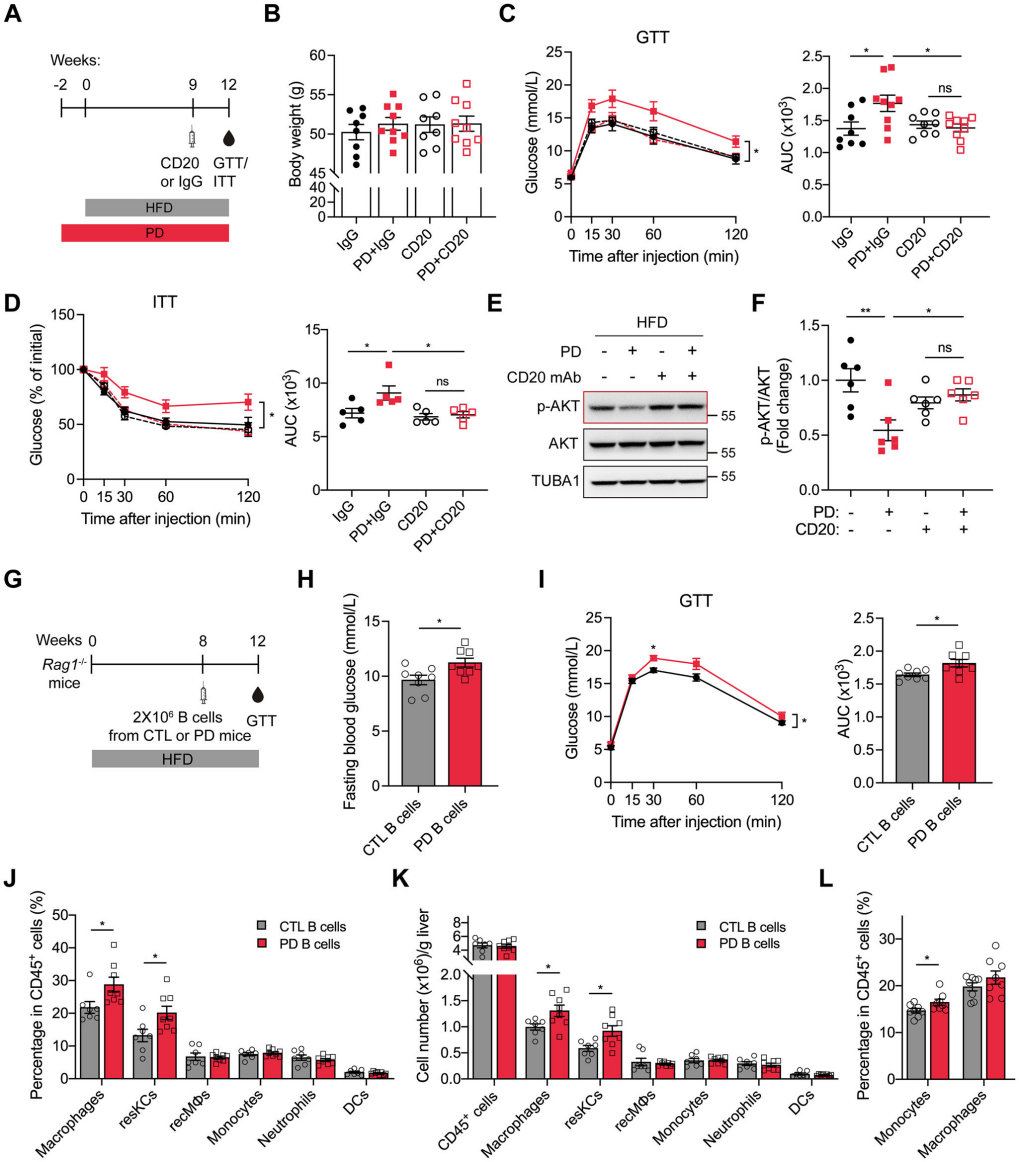
B cells mediate the metabolic disorder and resKC expansion elicited by PD in obese mice. (A) Schematic of the experimental design to investigate the effects of B cell depletion. CD20, a mouse CD20‐specific antibody. IgG served as an isotype control. (B) Body weight after 12 weeks of different treatments. (C) GTT and AUC analysis. (D) ITT and AUC analysis. (E) Representative immunoblots showing p‐AKT and total AKT in the livers of the four groups under basal conditions. (F) Densitometric quantification of the immunoblots exemplified in (E). (G) Schematic of the experimental design illustrating adoptive transfer of B cells from HFD or HFD+PD mice into *Rag*1^−^/^−^ mice. (H) Blood glucose levels after a 5 h fast. (I) GTT and AUC analysis. (J) Quantification of the indicated myeloid cell populations as percentages of CD45^+^ cells in the liver. (K) Quantification of the numbers of total CD45^+^ cells and the indicated hepatic myeloid cell populations. (L) Quantification of monocytes and macrophages as percentages of CD45^+^ cells in eWAT. Data are shown as mean ± SEM. Each data point represents an individual mouse. Two‐way ANOVA (left parts of C, D, and I), one‐way ANOVA (right parts of C and D; F), and unpaired Student's *t*‐test (H, J, K, and L; right part of I) were used for statistical analysis. ns, not significant. ^*^ *p* < 0.05, ^**^ *p* < 0.01.

To directly assess the role of B cells, obese mice were treated with a CD20‐specific antibody to systemically deplete B cells (Figure 5A). B cell depletion did not affect body weight (Figure 5B), but significantly improved glucose tolerance and insulin sensitivity in HFD+PD mice compared to IgG‐treated controls (Figure 5C,D). Given the pronounced hepatic responsiveness to PD, insulin signaling in the liver was next investigated. B cell depletion significantly improved hepatic insulin sensitivity in PD mice, as indicated by increased basal AKT phosphorylation in the absence of insulin stimulation (Figure 5E,F). To investigate whether PD remodeled B cell function, *Rag1^–/–^* mice (deficient in mature B and T cells [32]) were reconstituted with B cells isolated from HFD or HFD+PD donors (Figure 5G). B cells from HFD+PD mice recapitulated the metabolic impairments in recipient mice, including elevated fasting blood glucose levels and impaired glucose tolerance (Figure 5H,I). These phenotypes were accompanied by a significant expansion of hepatic macrophages, particularly resKCs, in both percentages and numbers (Figure 5J,K). Additionally, an increased percentage of monocytes and a modest but non‐significant increase in macrophages were observed in the eWAT (Figure 5L). These findings suggested that B cells mediated PD‐induced glucose dysregulation, and that hepatic macrophage accumulation could be attributed, at least in part, to the expansion of PD‐exposed B cells.

### 2.6 Interleukin‐18 Signaling Mediates PD‐Induced B Cell Proliferation and Differentiation

**FIGURE 6:**
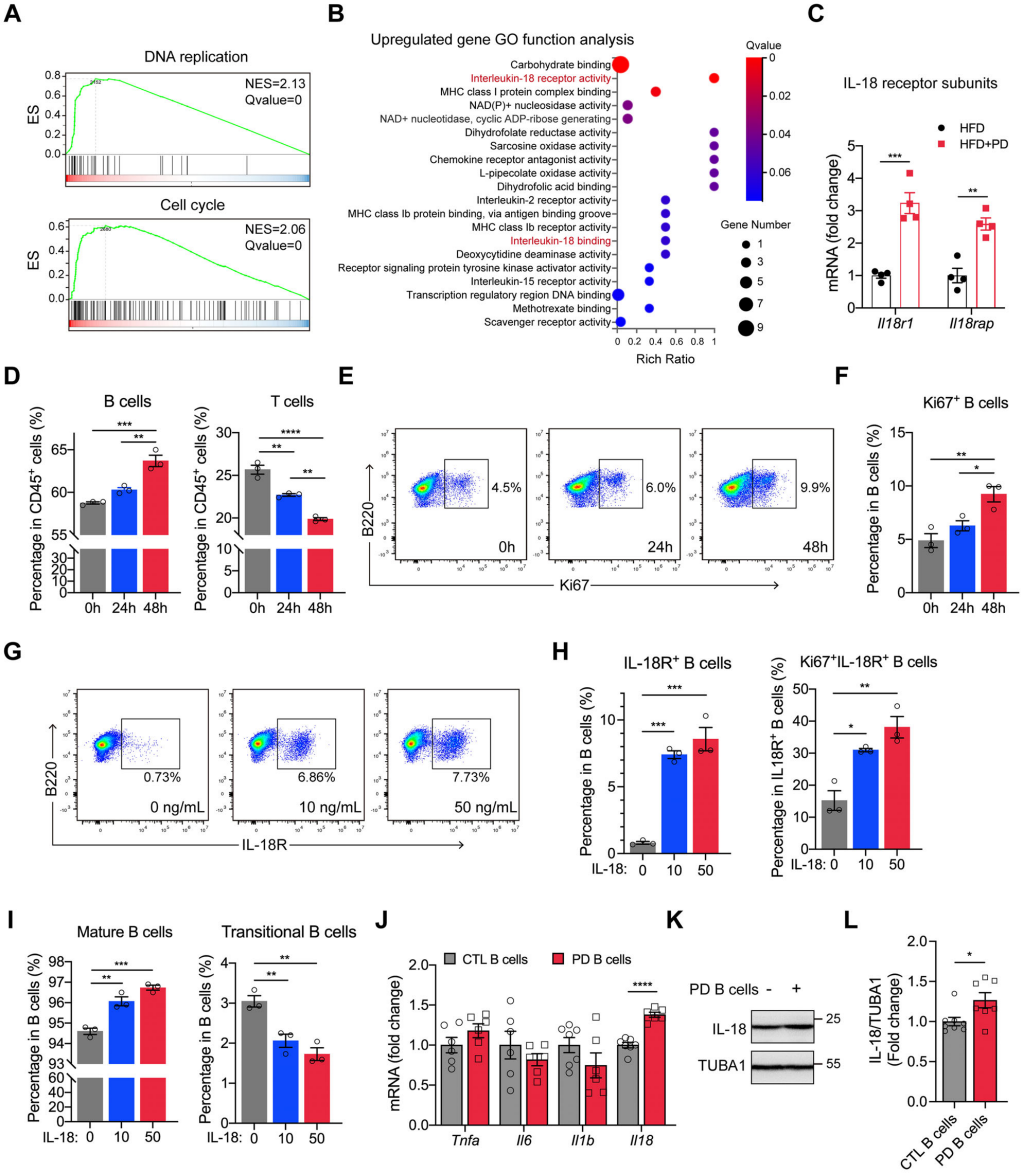
Interleukin (IL)‐18 signaling underlies PD‐induced expansion of B cells. (A) Gene set enrichment analysis of RNA‐seq data from splenic B cells (HFD+PD vs. HFD mice). ES, enrichment score; NES, normalized enrichment score. (B) Bubble plot showing Gene Ontology analysis of upregulated genes in HFD+PD vs. HFD mice. (C) qRT‐PCR analysis of *Il18r1* and *Il18rap* in splenic B cells. (D) Quantification of B and T cells as percentages of splenic CD45^+^ cells after stimulation with 50 ng/mL IL‐18 for 24 or 48 h. (E) Representative flow cytometry plots showing the proportions of proliferative (Ki67^+^) B cells among splenic B cells stimulated with IL‐18 (50 ng/mL) for different durations. (F) Quantification of Ki67^+^ B cells as percentages of total B cells. (G) Representative flow cytometry plots showing the proportions of IL‐18R^+^ B cells among splenic B cells stimulated with IL‐18 (0–50 ng/mL) for 48 h. (H) Quantification of IL‐18R^+^ B cells as percentages of total B cells (left) and Ki67^+^IL‐18R^+^ B cells as percentages of IL‐18R^+^ B cells (right) after IL‐18 stimulation. (I) Quantification of the indicated splenic B cell subsets as percentages of B cells after stimulation with IL‐18 (0–50 ng/mL) for 48 h. (J) qRT‐PCR analysis of inflammatory cytokines in the livers of *Rag1^–/–^* recipients after adoptive transfer of B cells from HFD or HFD+PD mice. (K) Representative immunoblots of IL‐18 in the livers of *Rag1* ^–/–^ recipients after B cell adoptive transfer. (L) Densitometric quantification of the immunoblots exemplified in (K). Data are shown as mean ± SEM. Unpaired Student's *t*‐test (C, J, and L) and one‐way ANOVA (D, F, H, and I) were used for statistical analysis. ^*^ *p* < 0.05, ^**^ *p* < 0.01, ^***^ *p* < 0.001, ^****^ *p* < 0.0001.

To elucidate how PD promoted B cell expansion, bulk RNA sequencing (RNA‐seq) was performed on splenic B cells isolated from HFD and HFD+PD mice. Gene set enrichment analysis revealed the enrichment of gene sets related to DNA replication and cell cycle pathways in B cells from HFD+PD mice (Figure 6A). Among the 60 significantly upregulated genes (Figure S10), Gene Ontology analysis revealed prominent enrichment of terms including carbohydrate binding, interleukin (IL)‐18 receptor activity, and MHC class I protein complex binding, with IL‐18 receptor activity showing the highest enrichment ratio (Figure 6B). The significant upregulation of IL‐18 receptor subunits, namely *Il18r1* and *Il18rap*, was validated by quantitative real‐time PCR (qRT‐PCR) (Figure 6C). Given the observed NLRP3 inflammasome activation and elevated IL‐18 levels in the liver and eWAT of HFD+PD mice (Figures 2, 3), we hypothesized that IL‐18 is implicated in PD‐induced B cell expansion. To test this hypothesis, splenocytes from HFD‐fed mice were treated with recombinant IL‐18 in vitro. IL‐18 increased the proportion of B cells and their proliferation in a time‐dependent manner (Figure 6D–F). IL‐18 also upregulated IL‐18R expression on B cells in a concentration‐dependent manner, suggesting the presence of a positive feedback loop (Figure 6G,H). The proportion of Ki67^+^IL‐18R^+^ B cells was significantly increased, indicating that the IL‐18‐responsive B cell population was proliferatively expanded (Figure 6H). Consistent with PD‐induced B cell maturation observed in vivo, IL‐18 promoted B cell maturation in vitro (Figure 6I). Moreover, PD‐exposed B cells enhanced hepatic IL‐18 expression in *Rag1^–/–^* recipients (Figure 6J–L), further supporting a positive feedback loop between B cell expansion and IL‐18, which was likely derived from macrophages due to resKC expansion. Together, these results demonstrated that IL‐18 signaling drove B cell proliferation and maturation in response to PD.

### 2.7 IL‐18 or Macrophage Depletion Attenuates PD‐Induced B Cell Expansion and Glucose Dysregulation

**FIGURE 7:**
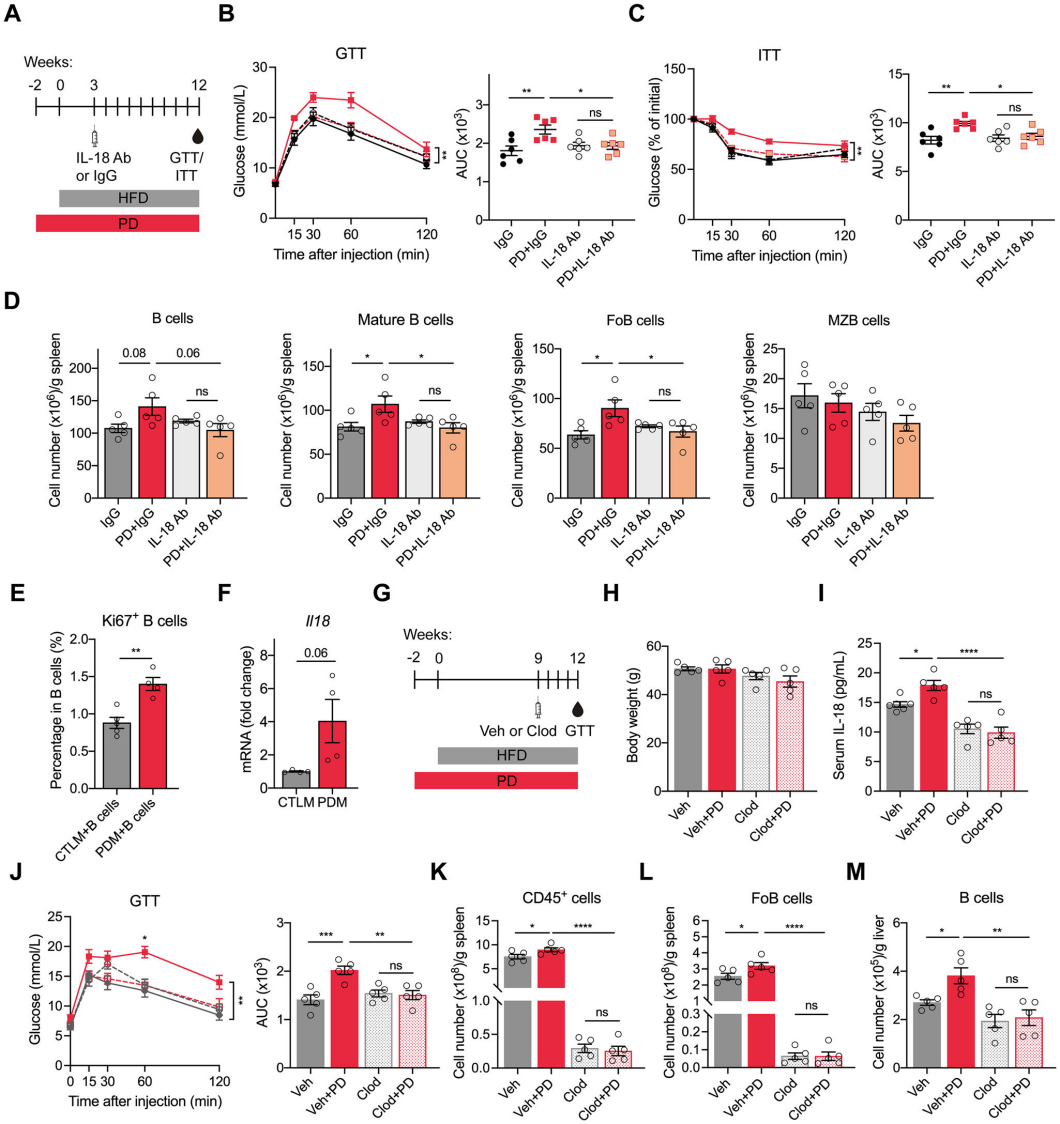
IL‐18 or macrophage depletion mitigates PD‐induced B cell expansion and glucose disorders. (A) Schematic of the experimental design to investigate the effects of IL‐18 neutralization. IL‐18 Ab, anti–IL‐18 antibody; IgG, isotype control. (B) GTT and AUC analysis. (C) ITT and AUC analysis. (D) Quantification of the numbers of B cells and major B cell subsets in the spleen. (E) Quantification of Ki67^+^ B cells as percentages of total B cells after co‐culture with hepatic macrophages isolated from HFD or HFD+PD mice for 48 h. CTLM, hepatic macrophages from HFD mice; PDM, hepatic macrophages from HFD+PD mice. (F) qRT‐PCR analysis of *Il18* in hepatic macrophages isolated from HFD or HFD+PD mice. Each point represents a pooled sample of cells from two mice within the same group. (G) Schematic of the experimental design to examine the effects of macrophage depletion. Clod, clodronate liposomes; Veh, vehicle liposomes. (H) Body weight after 12 weeks of different treatments. (I) Serum IL‐18 levels after 12 weeks of different treatments. (J) GTT and AUC analysis. (K) Quantification of splenic CD45^+^ cell numbers after liposome treatment. (L) Quantification of splenic FoB cell numbers normalized to tissue weight. (M) Quantification of hepatic B cell numbers normalized to tissue weight. Data are shown as mean ± SEM. Each data point represents an individual mouse, except for (E) and (F). Two‐way ANOVA (left parts of B, C, and J), one‐way ANOVA (right parts of B, C, and J; D, I, K, L, and M), and unpaired Student's *t*‐test (E, F) were used for statistical analysis. ns, not significant. ^*^ *p* < 0.05, ^**^ *p* < 0.01, ^***^ *p* < 0.001, ^****^ *p* < 0.0001.

To investigate the role of IL‐18 in the host response to PD, obese mice were treated with an IL‐18‐neutralizing antibody (Figure 7A). Compared with isotype controls, IL‐18 blockade significantly ameliorated PD‐induced metabolic disturbances, reduced splenic B cell numbers, and inhibited their maturation into FoB cells (Figure 7B–D). Given that macrophages are a vital source of IL‐18 [33], B cells were co‐cultured with hepatic macrophages isolated from HFD or HFD+PD mice. Hepatic macrophages from HFD+PD mice markedly enhanced B cell proliferation in vitro (Figure 7E). Consistently, qRT‐PCR analysis confirmed elevated *Il18* expression in macrophages derived from HFD+PD mice, identifying them as a key source of IL‐18 in the PD context (Figure 7F). Next, we investigated whether PD‐associated macrophages directly regulated B cell development. Macrophages were depleted by intravenous administration of clodronate liposomes, which have been shown to efficiently eliminate macrophages in the liver and spleen [34]. To minimize the weight loss and side effects caused by chronic treatment, we initiated liposome treatment at the late stage of HFD feeding (Figure 7G). Body weights were comparable across groups, although clodronate‐treated mice exhibited a modest decrease (Figure 7H). Macrophage depletion reduced serum IL‐18 levels in HFD mice and abrogated the PD‐induced IL‐18 surge (Figure 7I). Notably, macrophage depletion significantly reversed PD‐induced glucose intolerance (Figure 7J). Moreover, it led to a significant decrease in splenic immune cell numbers (Figure 7K), alleviation of PD‐induced B cell maturation (Figure 7L), and abolition of PD‐induced hepatic B cell accumulation (Figure 7M). Collectively, these results demonstrated that PD promoted macrophage–B cell crosstalk, likely mediated by IL‐18, to disrupt glucose homeostasis.

## 3 Discussion

**FIGURE 8:**
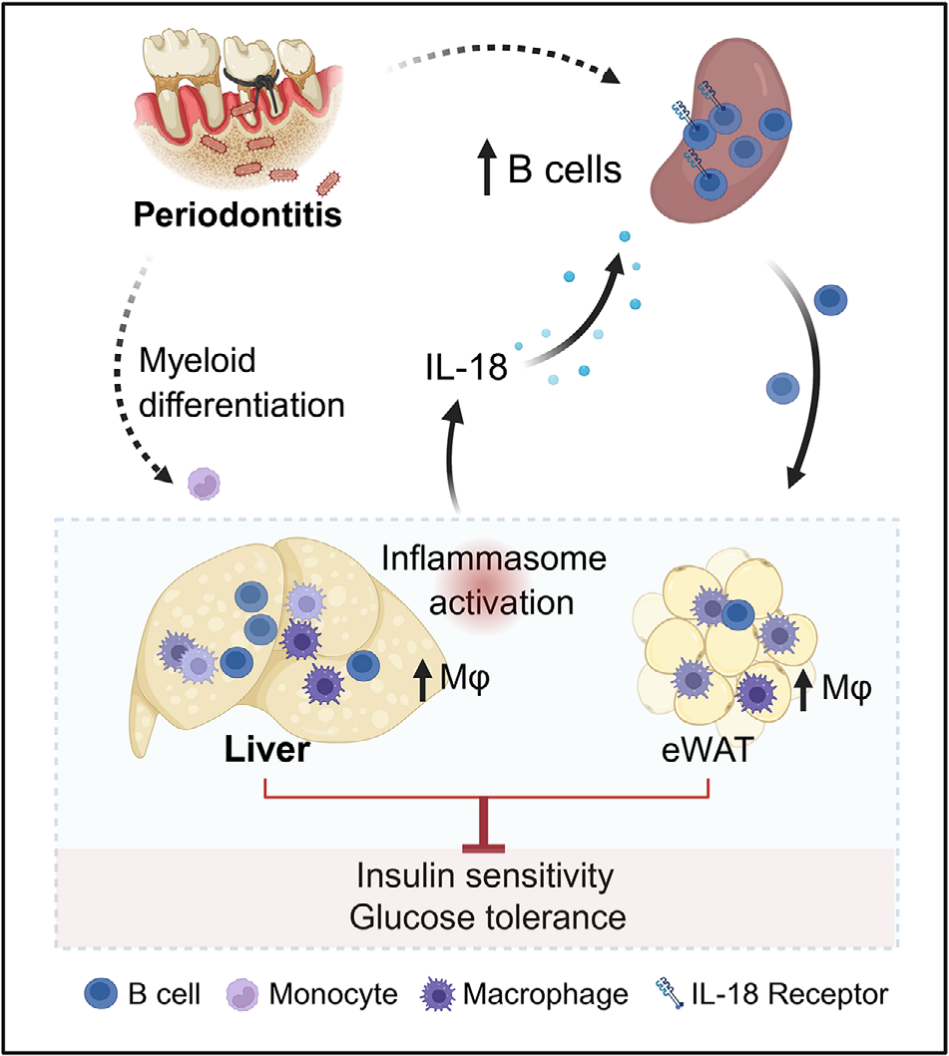
Proposed mechanism by which PD exacerbates glucose dysregulation in obesity. PD drives the pathogenic expansion of B cells and macrophages, thereby impairing glucose tolerance and insulin sensitivity in metabolic organs, particularly the liver. This effect is likely mediated by reciprocal crosstalk between macrophages and B cells via the NLRP3/IL‐18 signaling axis. The schematic illustration was created using BioRender.com.

Although epidemiological studies have established a bidirectional relationship between PD and T2D, the precise mechanisms through which PD exacerbates diabetic hyperglycemia remain unclear. In this study, we demonstrated that PD drove both local and systemic inflammation, thereby aggravating glucose dysregulation in a composite mouse model. A comprehensive characterization of the peripheral immune landscape revealed that B cells and macrophages were pivotal mediators of PD‐induced metabolic deterioration. Of note, these findings highlighted PD's regulation of aberrant immune cell interactions between pathogenic B cells and macrophages, in which NLRP3/IL‐18 signaling was involved (Figure 8).

Our data provide evidence that PD aggravated hyperglycemia and insulin resistance in obese mice. PD impaired insulin sensitivity in hepatic, adipose, and muscle tissues, with the most profound dysfunction observed in the liver. This tissue selectivity underscored the primacy of the oral−liver axis in mediating PD‐induced metabolic disturbances. Moreover, PD enhanced hepatic gluconeogenesis without significantly affecting hepatic steatosis, suggesting a preferential effect on glucose metabolism over lipid homeostasis. We therefore investigated the immunological basis of these metabolic alterations, in which both innate and adaptive immune responses were implicated.

Existing mouse models deciphering the causality between PD and diabetes often rely on oral inoculation of specific pathogens [35, 36] or periodontal injection of lipopolysaccharide (LPS) [37] in diabetic animals induced by streptozotocin [36] or genetic mutation [38]. In contrast, the combination of long‐term ligature‐induced PD and HFD‐induced diabetes has been less well explored. Excessive body fat accumulation is a critical contributor to T2D [1], and diet‐induced obese mouse models recapitulate most key features of human diabetes. Additionally, the PD model used in this study mirrors the chronic, dysbiotic nature of human PD [39]. Although PD increased tooth mobility in obese mice, we circumvented the adverse effect of loose teeth on food intake, which could have otherwise influenced the outcome of diabetes. PD induced multiorgan insulin resistance along with chronic low‐grade inflammation, manifested by pro‐inflammatory macrophage infiltration as well as NLRP3 inflammasome activation.

Macrophages, central players in the innate immune system, are well‐established contributors to insulin resistance and unresolved inflammation [23]. Macrophages comprise heterogeneous populations with specialized functions across different tissue niches [40]. The liver, in particular, harbors the largest population of macrophages under steady‐state conditions [22]. Both resKCs and recMφs contribute to impaired hepatic insulin sensitivity [41]. Our findings highlighted the functional differences of these subsets in the context of obesity and PD, with resKCs showing a particular responsiveness to PD and involvement in B cell‐mediated immune responses. Previous studies have shown that in steatotic livers, resKCs store lipids [42], subsequently lose their self‐renewal capacity, and undergo cell death [43]. Circulating monocytes can then enter the vacant niches left by dying resKCs, acquire Kupffer cell‐specific identity, and become monocyte‐derived resKCs [44, 45]. In the case of bacterial infections, resKCs replenish their population and restore scavenger and phagocytic functions, which may explain the increase in recMφs and resKCs during periodontal infection.

Beyond classical roles in humoral immunity, B cells have recently been recognized as determinants in the pathogenesis of T2D [27]. For instance, obese mice deficient in mature B cells exhibit reduced systemic inflammation, improved insulin sensitivity, and increased regulatory T cells compared with their wild‐type counterparts [46]. A similar study has demonstrated that B cells exert detrimental metabolic effects through T cell modulation and IgG antibody production [47]. While previous work has shown that CD20‐mediated B cell depletion attenuates obesity‐related glucose abnormalities, no significant differences were observed between CD20‐treated and untreated obese mice in our study. This discrepancy may be attributed to differences in mouse age, body weight, and the timing of HFD feeding. Specifically, B cells comprise at least two distinct lineages: B1 and B2 cells [48]. As a major circulating population, B2 cells give rise to plasma or memory B cells in the periphery [49]. In humans, B cell subset imbalances have been observed in obesity [50], and B2 cells positively correlate with both glycemia and lipidemia [51]. Consistently, our results suggested that the pathogenic mobilization of B2 cells elicited by PD promoted glucose dysregulation. In contrast, B1 cells confer protection against insulin resistance via IL‐10 secretion or IgM‐dependent mechanisms [52, 53].

Our study also uncovered a distinct interaction between B cells and macrophages, particularly resKCs, that was driven by PD. This aligns with findings in other chronic diseases, where communication between disease‐relevant B cells and macrophages propagates neuroinflammation [54]. Tissue‐resident B cells can regulate macrophage polarization and function [55]. We demonstrated that PD‐relevant B cells were sufficient to drive macrophage expansion and, in turn, stimulate their production of IL‐18. Conversely, macrophages exposed to the PD microenvironment enhanced B cell proliferation, thereby establishing a self‐reinforcing inflammatory circuit. This PD‐triggered, bidirectional crosstalk served as a key amplifier of metabolic inflammation in obesity. Mechanistically, such immune crosstalk can be orchestrated by receptor‐ligand interactions or cytokines [56]. For example, peritoneal macrophages promote B1 cell survival through IL‐6 secretion [57], whereas type I interferons and B cell‐activating factors mediate communication in other contexts [56]. Our results identified IL‐18 as a central mediator of this pathogenic crosstalk. The upregulation of IL‐18R on PD‐exposed B cells, coupled with our functional data, suggested the essential role of IL‐18 signaling in driving B cell expansion and promoting their pro‐inflammatory synergy with macrophages.

IL‐18 first emerges as a macrophage‐derived cytokine whose production can be induced by inflammatory stimuli (e.g., LPS and interferons) [58]. In macrophages, NOD‐like receptor activation triggers NLRP3 inflammasome assembly, leading to caspase‐1‐dependent production of IL‐1β and IL‐18 [59]. Obesity upregulates NLRP3 expression, which instigates metabolic inflammation and insulin resistance [60]. Clinically, circulating IL‐18 levels are elevated in obese individuals and decline after weight loss intervention [61], further implicating IL‐18 in the pathogenesis of metabolic syndrome [62]. In our study, PD reinforced NLRP3 inflammasome activation, potentially driven by microbial perturbation and the systemic dissemination of microbial products, which consequently led to the elevation of systemic IL‐18. Consistently, macrophages derived from HFD+PD mice secreted higher levels of IL‐18 compared to those from controls. Upon binding to its receptor complex, mature IL‐18 activates downstream signaling pathways including NF‐κB [63]. *Il18r1* and *Il18rap* expression in B cells has been reported but is of uncertain significance [58, 64]. In this regard, our results elucidated the function of IL‐18/IL‐18R signaling in B cell proliferation, presenting a potential avenue for cytokine‐directed therapies to dampen the inflammatory response in PD and related comorbidities, including T2D.

While our study systematically delineated the impact of PD on the immune landscape in obesity, several intriguing findings warrant further investigation. For instance, the expansion of neutrophils was observed in the liver and eWAT of HFD+PD mice. Furthermore, despite comparable total T cell numbers in the liver, PD exposure induced a consistent and preferential reduction in the percentage of CD4^+^ T cells over CD8^+^ T cells across the liver, blood, and spleen. *Rag1* ^−^/^−^ mice were used to minimize the potential confounding influence of endogenous T cells when interpreting the phenotypic consequences of adoptively transferred B cells. However, the specific regulatory roles of T cell subsets, particularly the skewed CD4/CD8 ratio, remain to be elucidated.

In summary, our study establishes a causal relationship between PD and T2D progression from metabolic and immunological perspectives. Mechanistically, PD drives the systemic expansion of pathogenic B cells that impairs glucose homeostasis, which is likely achieved through crosstalk between macrophages and B cells via the NLRP3/IL‐18 signaling axis. Targeting these mechanisms may rescue the aberrant immune cell interactions and metabolic sequelae of PD.

## 4 Methods

### 4.1 Animals

The study protocol was approved by the Institutional Review and Ethics Board of Shanghai Ninth People's Hospital, Shanghai Jiao Tong University School of Medicine (approval no. SH9H‐2021‐A1041‐1 and SH9H‐2021‐A1064‐1) and complied with the institutional guidelines. Eight‐week‐old male C57BL/6 and *Rag1^–/–^* mice were obtained from Beijing Vital River Laboratory Animal Technology Co., Ltd. (Beijing, China) and GemPharmatech (Nanjing, China), respectively. All mice were maintained under specific pathogen‐free conditions with ad libitum access to food and water on a 12:12 h light: dark cycle. Mice from different cages were co‐housed for two weeks to homogenize their commensal microbiota before the initiation of experiments.

### 4.2 In Vivo Mouse Models

Fourteen‐week‐old mice were subjected to long‐term ligation or a sham operation under anesthesia. Ligation was performed as previously described [19]. Briefly, bilateral maxillary second molars were ligated using 5–0 silk sutures. The sham operation involved the insertion and subsequent removal of silk sutures after 12 h. Two weeks post‐operation, mice were fed either HFD (D12492, Research Diets) or NCD for 12 weeks. Ligatures were retained in the PD group throughout the experimental period. For B cell depletion, a single intravenous injection of an anti‐CD20 antibody (152116, BioLegend) or isotype control (400672, BioLegend) (250 µg/mouse) was administered via the tail vein after 9 weeks of HFD feeding, and mice were subsequently maintained on HFD for an additional 3 weeks. To deplete macrophages, clodronate liposomes or control liposomes (CP‐010‐010, Liposoma) were administered intravenously (200 µL/mouse) via the tail vein every 5 days, beginning at week 9 of HFD feeding and continuing until euthanasia at week 12. For IL‐18 neutralization, mice were intraperitoneally injected with an anti‐IL‐18 antibody (BE0237, BioXCell) or isotype controls (BE0089, BioXCell) (100 µg/mouse) once weekly, starting at week 3 of HFD feeding and continuing until euthanasia at week 12.

### 4.3 Assessment of Alveolar Bone Loss

Formalin‐fixed maxillae were defleshed in 5% sodium hypochlorite and bleached in 5% hydrogen peroxide. The specimens were then stained with 1% methylene blue and photographed using a stereomicroscope (Leica). To quantify alveolar bone loss, the distance from the cementoenamel junction to the alveolar bone crest was measured on the palatal side of the maxillary molars. The following six sites were selected for measurement [19]: the distal groove and distal cusp of the first molar; the mesial cusp, groove, and distal cusp of the second molar; and the cusp of the third molar.

### 4.4 Cell Preparation for Flow Cytometry

To generate single‐cell suspensions, spleens were mechanically mashed through 70 µm cell strainers using a syringe plunger and rinsed with ice‐cold phosphate‐buffered saline supplemented with 2% fetal bovine serum (FBS). Blood samples were collected into EDTA‐coated tubes. Hepatic mononuclear cells (MNCs) were isolated by enzymatic digestion as previously described [65]. Briefly, mouse livers were dissociated using a gentleMACS Dissociator (Miltenyi Biotec) and digested in Hanks' Balanced Salt Solution (HBSS) (B548143, Sangon Biotech) supplemented with 0.75 mg/mL type IV collagenase (A004186, Sangon Biotech) at 37°C for 30 min on a rotating shaker, followed by filtration through 70 µm cell strainers. After centrifugation, liver homogenates were subjected to 40%/80% Percoll (17‐0891‐01, Cytiva) gradient separation to isolate leukocytes. Stromal vascular cells (SVCs) from adipose tissue were prepared as previously described [66]. Briefly, adipose tissue was minced and digested in HBSS containing 1 mg/mL type II collagenase (A004174, Sangon Biotech) at 37°C for 20 min on a rotating shaker. After filtration, the SVCs were pelleted by centrifugation. Red blood cells were lysed prior to antibody staining procedures.

### 4.5 Flow Cytometry

Single‐cell suspensions were first stained with a viability dye (565694 or 564996, BD Pharmingen) for 15 min at room temperature. Cells were then washed and incubated with an Fc receptor blocking antibody (101320, BioLegend) for 10 min on ice, followed by incubation with cell surface‐staining antibodies for 30 min on ice in the dark. For intracellular targets (e.g., CD206), cells were fixed and permeabilized using an intracellular staining kit (88‐8824, Invitrogen) prior to antibody incubation. For nuclear proteins (e.g., Ki67), a transcription factor staining buffer set (00‐5523, Invitrogen) was used. Finally, stained cells were analyzed on a BD LSRFortessa X‐20 cell analyzer (BD Biosciences) or sorted using a BD FACSAria III cell sorter (BD Biosciences). Data were analyzed using FlowJo software (FlowJo LLC).

The following antibodies were used in flow cytometry: FITC anti‐CD45 (103108), PE/Cyanine7 anti‐CD11b (101216), PerCP‐Cy5.5 anti‐CD45 (103131), and PE anti‐CD218a (IL‐18Rα) (157904) from BioLegend; APC‐Cy7 anti‐CD45 (557659), PerCP‐Cy5.5 anti‐Ly‐6G (560602), FITC anti‐CD11b (557396), PE‐Cy7 anti‐Ly‐6C (560593), BUV737 anti‐CD11c (612796), BV421 anti‐I‐A/I‐E (562564), PE anti‐F4/80 (565410), Alexa Fluor 647 anti‐CD206 (565250), FITC anti‐CD3e (553061), APC anti‐CD45R/B220 (561880), PerCP‐Cy5.5 anti‐CD8a (561109), PE anti‐CD4 (553652), PE‐Cy7 anti‐NK‐1.1 (562062), APC‐Cy7 anti‐CD19 (557655), PE anti‐CD23 (561773), BV421 anti‐CD43 (562958), BB700 anti‐CD5 (742083), FITC anti‐CD45R/B220 (553087), BV605 anti‐CD93 (740386), PE‐Cy7 anti‐IgM (552867), APC anti‐CD21/CD35 (561770), and BV421 anti‐Ki‐67 (562899) from BD Pharmingen.

### 4.6 B Cell Isolation and Adoptive Transfer

B cells were isolated from the spleens of control and PD mice after 12 weeks of HFD feeding and designated as CTL B and PD B cells, respectively. Splenic homogenates were subjected to 40%/80% Percoll gradient separation to isolate leukocytes. B cells were then purified by negative selection using a commercial B cell isolation kit (130‐104‐443, Miltenyi Biotec) according to the manufacturer's protocol. The purity of the isolated B cells (93%) was confirmed by flow cytometry. A total of 2 × 10^6^ B cells were intraperitoneally injected into 20‐week‐old male *Rag1* ^–/–^ recipient mice that had been fed an HFD for 8 weeks. Experiments were performed 4 weeks after adoptive transfer as previously described [67].

### 4.7 Metabolic Tolerance Tests

Metabolic tolerance tests were performed as previously described with minor modifications [68]. Mice in each group were housed in individual cages, free from uncontrolled external stimuli such as human traffic or unnecessary noise. To prevent injection‐related stress from biasing comparisons across groups, all the mice were administered identical intraperitoneal injections by the same experienced researcher. For the glucose tolerance test, mice were fasted for 15 h and then challenged with an intraperitoneal injection of glucose (1 g/kg). For the insulin tolerance test, mice were fasted for 5 h prior to an intraperitoneal injection of insulin (HI‐710, Lilly France) (1 U/kg). For the PTT, mice were fasted for 15 h and received an intraperitoneal injection of sodium pyruvate (1 g/kg). Blood glucose levels were measured in the tail vein blood at designated time points using a glucometer (Abbott Laboratories). The area under the curve over the 0–120 min period was calculated as a comprehensive measure, with a focus on the overall response for each test.

### 4.8 Acute Insulin Stimulation and Tissue Collection

After a 5 h fast, mice were injected intraperitoneally with insulin (5 U/kg) or an equal volume of saline as a control. The liver, eWAT, sWAT, and soleus muscle were collected 10 min after injection and snap‐frozen in liquid nitrogen. Samples were stored at −80°C until processing.

### 4.9 Serum Biochemical Assays

Fasting serum insulin levels were quantified using a Mouse Ultrasensitive Insulin ELISA Kit (80‐INSMSU‐E01, ALPCO) according to the manufacturer's instructions. Fasting blood glucose levels were measured using a glucometer. Serum AST, ALT, triglycerides, and cholesterol levels were assessed using an automated biochemistry analyzer (Hitachi). The serum IL‐18 concentration was measured using an ELISA kit (EMC011, Neobioscience).

### 4.10 Hepatic Triglyceride Quantification

Hepatic triglyceride content was determined using a Triglyceride Enzymatic Assay Kit (E1013, Applygen) following the manufacturer's instructions.

### 4.11 qRT‐PCR

Total RNA was extracted from the liver, sWAT, eWAT, B cells, and macrophages using TRIzol reagent (15596‐018, Thermo Fisher Scientific). RNA concentration and purity were assessed using a NanoDrop spectrophotometer (Thermo Fisher Scientific). Complementary DNA (cDNA) was synthesized using the PrimeScript RT reagent kit (RR037A, TaKaRa). qRT‐PCR was performed using cDNA, TB Green Premix (RR420A, TaKaRa), and gene‐specific primers (Table S1) on a LightCycler 480 System (Roche). Relative gene expression was normalized to *Gapdh* expression, and fold changes were calculated using the ∆∆Ct method.

### 4.12 Western Blotting

Frozen tissues were homogenized and lysed in a cell lysis buffer (P0013, Beyotime) supplemented with protease and phosphatase inhibitors (B14001 and B15001, Selleck). The protein concentration in the supernatant was quantified using a BCA assay kit (23225, Thermo Fisher Scientific). Equal amounts of protein were separated by sodium dodecyl sulfate‐polyacrylamide gel electrophoresis and transferred onto polyvinylidene fluoride membranes. After blocking with 5% non‐fat milk for 1 h, membranes were incubated with primary antibodies overnight at 4°C. Membranes were then incubated with species‐appropriate secondary antibodies. Protein bands were visualized using a chemiluminescent substrate (34580, Thermo Fisher Scientific) and captured on a Fusion imaging system (Vilber).

The following primary antibodies were used: p‐AKT (Ser473; 9271), AKT (9272), and IL‐1β (12242) from Cell Signaling Technology; TUBA1 (T6199) from Sigma–Aldrich; NLRP3 (AG‐20B‐0014), CASP1 (AG‐20B‐0042), and ASC (AG‐25B‐0006) from Adipogen; and IL‐18 (ab191860) from Abcam.

### 4.13 Histology and Immunohistochemistry Analyses

Liver and adipose tissues were fixed in 4% paraformaldehyde, embedded in paraffin, and sectioned at a thickness of 5 µm. Maxillae were fixed and decalcified before paraffin embedding. Deparaffinized sections were stained with a H&E commercial kit (BL700B, Biosharp). For immunohistochemistry, deparaffinized sections were treated with a citric acid antigen retrieval solution (P0086, Beyotime) and then blocked with 5% normal goat serum. Sections were incubated with a Mac‐2 primary antibody (14‐5301‐81, Invitrogen) overnight and then with the appropriate horseradish peroxidase‐conjugated secondary antibodies. A DAB detection kit (G1212, Servicebio) was used to visualize antibody binding, and the nuclei were counterstained with hematoxylin.

### 4.14 Cell Experiments

Macrophages were isolated from the hepatic MNCs of HFD and HFD+PD mice by fluorescence‐activated cell sorting with a cocktail of CD45, CD11b, and F4/80 antibodies. B cells were isolated from the spleens of untreated mice as described above. Co‐culture experiments were performed as previously described, with minor modifications [69]. Briefly, macrophages and B cells were seeded in 96‐well plates at a 1:3 ratio with a total density of 2 × 10^5^ cells per well in DMEM/F12 medium (11320033, Gibco) supplemented with 10% FBS and 1% penicillin‐streptomycin. After 48 h of co‐culture, B cells were collected for flow cytometric analysis. For IL‐18 stimulation, splenocytes from HFD mice were cultured at 3 × 10^6^ cells/mL in complete RPMI 1640 medium (C11875500BT, Gibco). Cells were treated with recombinant mouse IL‐18 protein (767004, BioLegend) at the indicated concentrations (0, 10, 50 ng/mL) for 0, 24, or 48 h as specified in the figure legends.

### 4.15 RNA Sequencing and Bioinformatic Analysis

B cells were purified from splenic single‐cell suspensions derived from HFD and HFD+PD mice using a B cell isolation kit. Total RNA was extracted using TRIzol reagent. RNA‐seq was performed on the BGISEQ platform (BGI, China), and the reads were mapped to the mouse genome (*Mus musculus*, GCF_000001635.26_GRCm38.p6). Data analysis and visualization were performed using the Dr. Tom multi‐omics data mining system provided by BGI (https://biosys.bgi.com). A *Q*‐value ≤ 0.05 and fold change > 1.5 were set as thresholds for significant differential expression.

### 4.16 Statistical Analysis

Statistical analyses were performed using GraphPad Prism (GraphPad Software). Data are presented as mean ± SEM. For comparisons between two groups, two‐tailed unpaired Student's *t*‐tests were used. For comparisons among more than two groups, one‐way ANOVA followed by Tukey's multiple comparison tests was used. For repeated‐measures data, two‐way ANOVA followed by Bonferroni's multiple comparison tests was used. Statistical significance was defined as follows: ^*^ *p* < 0.05, ^**^ *p* < 0.01, ^***^ *p* < 0.001, and ^****^ *p* < 0.0001.

## Author Contributions

S.‐Z.D., Y.‐Q.Z., L.‐J.Z., and W.‐Z.L. contributed to conceptualization. Funding acquisition was carried out by S.‐Z.D., Y.‐Q.Z., and W.‐Z.L. Methodology was developed by W.‐Z.L., L.‐J.Z., T.L., L.‐J.D., Y.L., Y.‐L.W., H.Z., Y.L., and W.‐C.Z. Project administration was performed by W.‐Z.L., L.‐J.Z., H.‐L.Y., T.L., B.‐Y.C., X.‐B.B., J.Z., L.B., Y.‐L.L., S.X., X.‐Q.M., and G.‐C.T. Visualization was completed by W.‐Z.L., T.L., L.‐J.Z., and H.‐L.Y. Supervision was provided by S.‐Z.D., W.‐C.Z., and Y.‐Q.Z. The original draft was written by W.‐Z.L. and S.‐Z.D., while review and editing were conducted by W.‐Z.L., L.‐J.Z., H.‐L.Y., T.L., B.‐Y.C., X.‐B.B., J.Z., L.B., L.‐J.D., Y.L., Y.‐L.W., H.Z., Y.‐L.L., S.X., X.‐Q.M., G.‐C.T., Y.L., W.‐C.Z., Y.‐Q.Z., and S.‐Z.D.

## Funding

This work was supported by grants from the National Natural Science Foundation of China (82330015, 82370953, 81991503, 81991500), the National Key Research and Development Program of China (2023YFA1801100, 2023YFA1801104), the Zhejiang Provincial Leading Innovation and Entrepreneurship Team (2024R01003), and the China Postdoctoral Science Foundation (2023M742319).

## Conflicts of Interest

The authors declare no conflicts of interest.

## Supporting information




**Supporting File**: advs74234‐sup‐0001‐SuppMat.pdf.

## Data Availability

The data that support the findings of this study are available from the corresponding author upon reasonable request.
