# Supplementary material for: Periodontitis Induces B Cell‐Macrophage Crosstalk to Exacerbate Glucose Dysregulation in Obesity
Source: Adv Sci (Weinh). 2026 Mar 20;13(23):e17653. doi: 10.1002/advs.202517653 (PMC13104131; doi:10.1002/advs.202517653)
Supplement: Supplementary file 1 — Supporting File: advs74234‐sup‐0001‐SuppMat.pdf. [file ADVS-13-e17653-s001.pdf]

Periodontitis Induces B Cell-Macrophage Crosstalk to Exacerbate Glucose Dysregulation in Obesity

Wen-Zhen Lin[#], Lu-Jun Zhou[#], Hui-Lin Ye[#], Ting Liu, Bo-Yan Chen, Xue-Bing Bai, Jun Zhang, Lan Bai, Lin-Juan Du, Yuan Liu, Yong-Li Wang, Hong Zhu, Yu-Lin Li, Shuo Xu, Xiao-Qian Meng, Guo-Cai Tian, Yan Liu, Wu-Chang Zhang, Ya-Qin Zhu[*], Sheng-Zhong Duan[*]

Supporting Information

Supplemental Figures

Figure S1. PD promotes alveolar bone resorption in mice.

Figure S2. PD does not affect weight distribution or food consumption in mice.

Figure S3. PD does not affect lipid metabolism in mice.

Figure S4. Flow cytometry gating strategy for lymphocytes.

Figure S5. Flow cytometry gating strategy for myeloid cells in the liver.

Figure S6. Analysis of immune cell infiltration in adipose tissue.

Figure S7. Flow cytometry gating strategy for myeloid cells in the spleen.

Figure S8. Flow cytometric analysis for B cell subsets in the periphery.

Figure S9. Flow cytometric analysis for B cell maturation in the spleen.

Figure S10. Differential gene expression analysis of splenic B cells from HFD+PD vs. HFD mice.

Supplemental Tables

Table S1. Primer sequences for qRT-PCR.

Figure S1

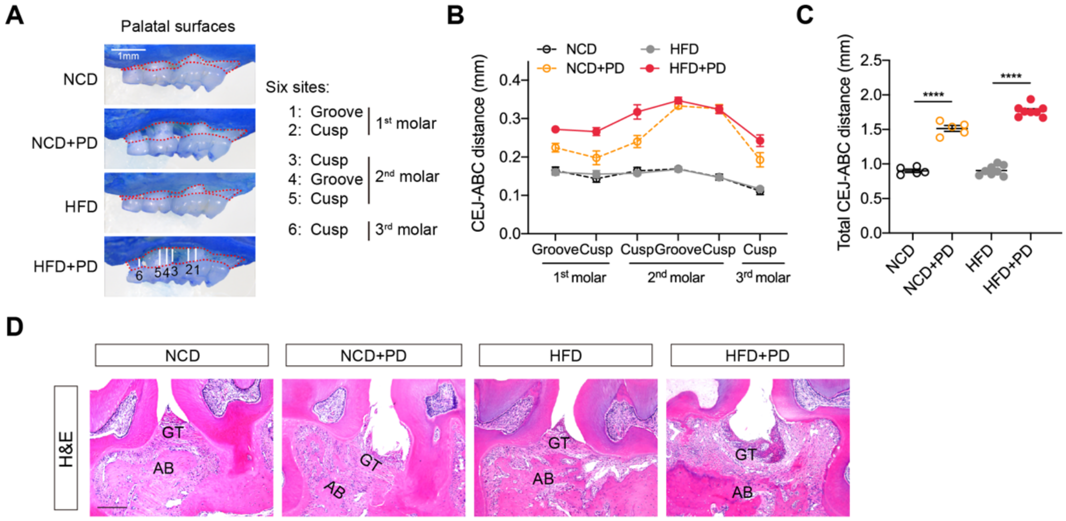

Figure S1. PD promotes alveolar bone resorption in mice. (A) Palatal views of maxillae from mice treated with or without (±) PD and fed either an NCD or HFD for 12 weeks. (B) Measurements of the distance from the cementoenamel junction to the alveolar bone crest (CEJ–ABC) on the palatal side of maxillary molars, indicating periodontal bone loss. (C) Total six-site CEJ–ABC distance on the palatal side of maxillary molars. (D) Hematoxylin and eosin (H&E) staining of cross-sections of maxillae. GT, gingival tissue; AB, alveolar bone. Scale bar, 200 µm. Data are presented as mean ± SEM. Unpaired Student's *t*-test was used for statistical analysis. $^{****}p < 0.0001$.

Figure S2

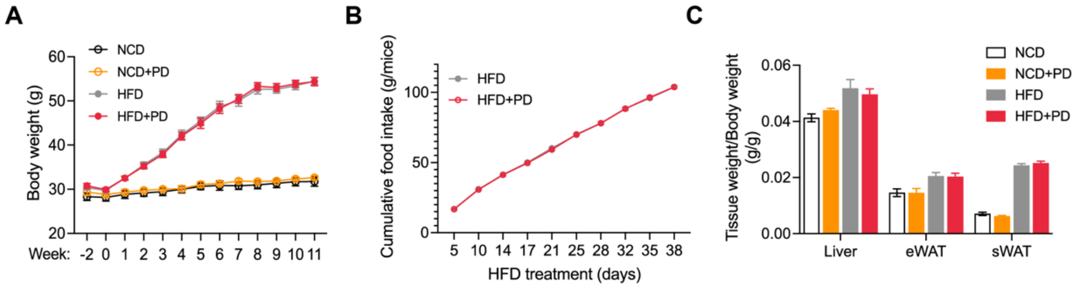

Figure S2. PD does not affect weight distribution or food consumption in mice. (A) Body weights of lean and obese mice (n = 5–8 per group). (B) Cumulative food intake in obese mice (n = 5 per group). (C) Weights of the liver, eWAT, and sWAT, normalized to body weight (n = 7–10 per group). Data are presented as mean ± SEM.

Figure S3

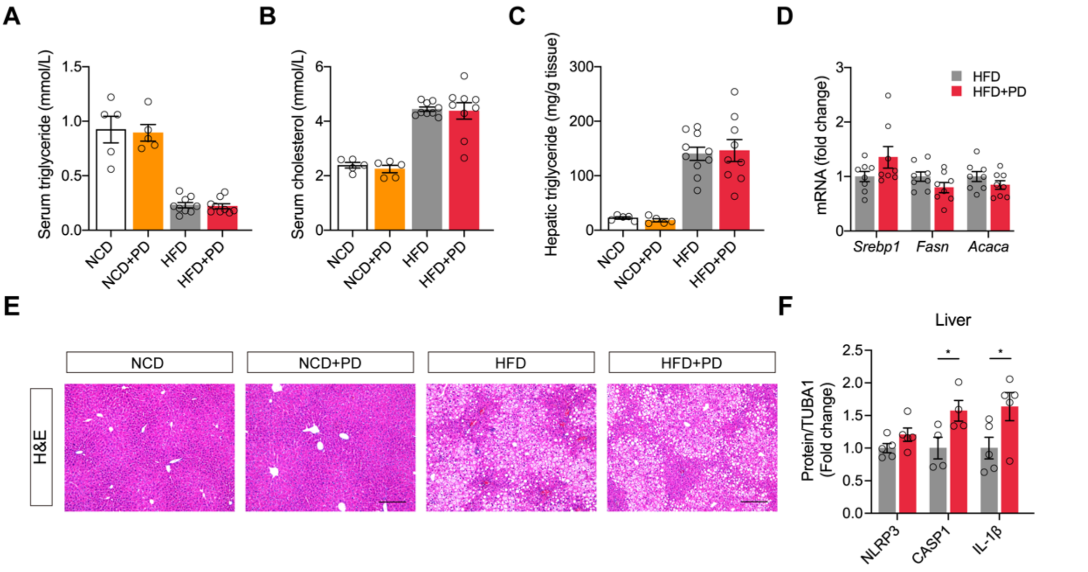

Figure S3. PD does not affect lipid metabolism in mice. (A) Serum triglyceride levels. (B) Serum cholesterol levels. (C) Hepatic triglyceride content normalized to tissue weight. (D) qRT-PCR analysis of lipogenic genes in the liver of obese mice. (E) Representative H&E-stained liver sections. Scale bar, 200 µm. (F) Densitometric quantification of the immunoblots exemplified in Figure 2D. Data are presented as mean ± SEM. Each data point represents an individual mouse. Unpaired Student's *t*-test was used for statistical analysis. $^{*}p < 0.05$.

Figure S4

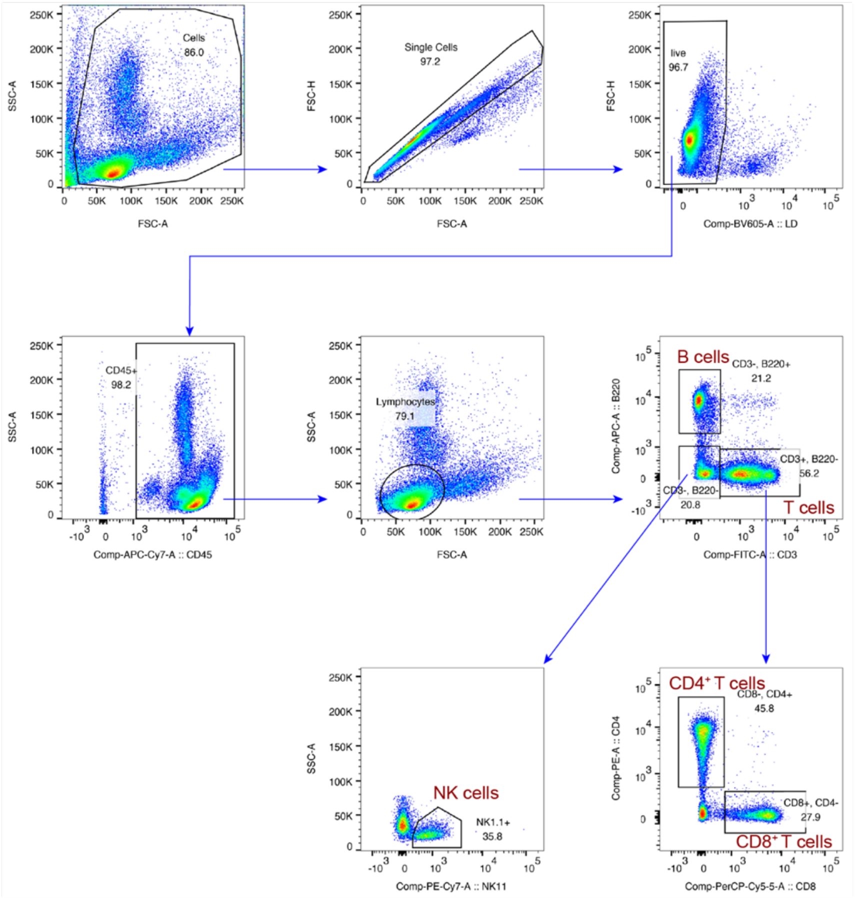

Figure S4. Flow cytometry gating strategy for lymphocytes. After excluding debris, doublets, and dead cells, $CD45^+$ cells were selected. Lymphocytes were identified based on FSC/SSC and stratified into T cells and B cells via CD3 and B220 gating. T cells were further divided into $CD8^+$ T cells and $CD4^+$ T cells. NK cells were gated by the expression of NK1.1 and the absence of CD3 and B220.

Figure S5

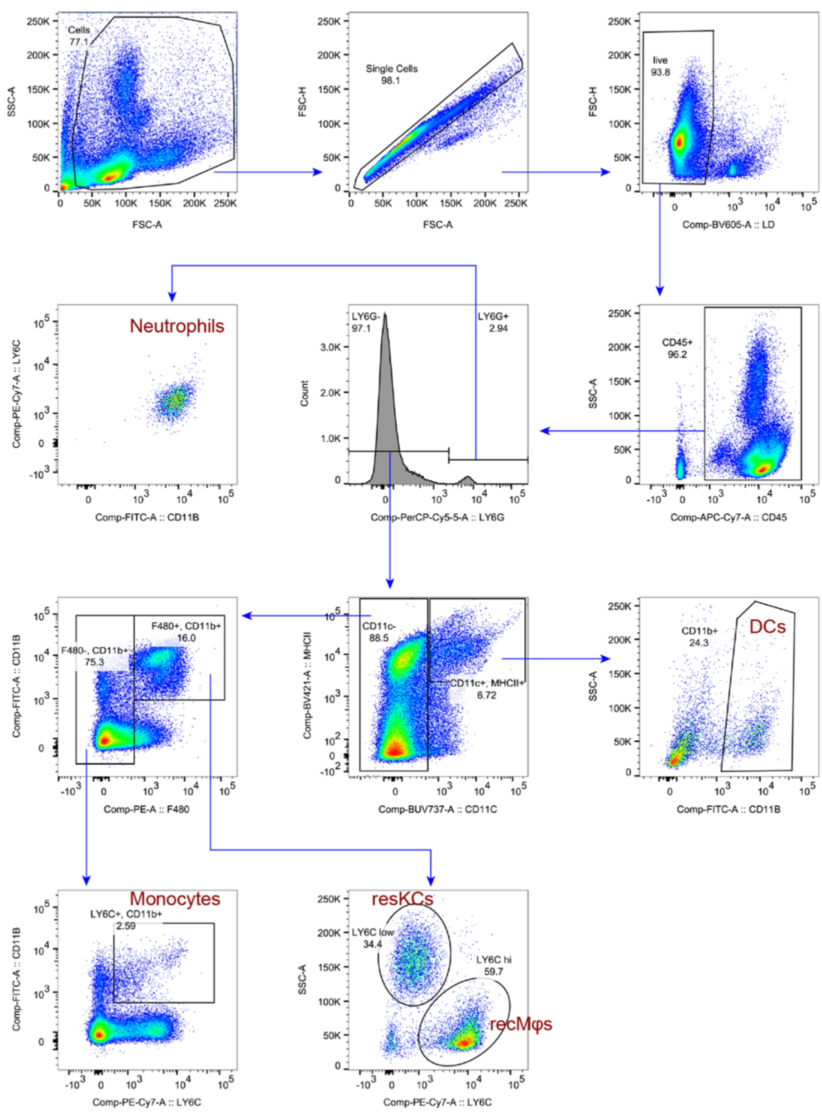

Figure S5. Flow cytometry gating strategy for myeloid cells in the liver. After gating for singlets, live cells, and CD45^{+} cells, hepatic neutrophils were identified as Ly6G^{+}CD11b^{+}Ly6C^{int} cells. The non-neutrophil (Ly6G^{-}) population was then subdivided based on CD11c and MHCII expression. The CD11c^{+}MHCII^{+}CD11b^{+} population was gated as DCs. The CD11c^{-} cell population was subsequently classified via F4/80 and CD11b gating. Among F4/80^{+}CD11b^{+} cells, hepatic macrophages were identified as Ly6C^{lo} resKCs with high SSC, and Ly6C^{hi} recMφs. Monocytes were then gated from the F4/80^{-} subpopulation, which were Ly6C^{+}CD11b^{+}.

Figure S6

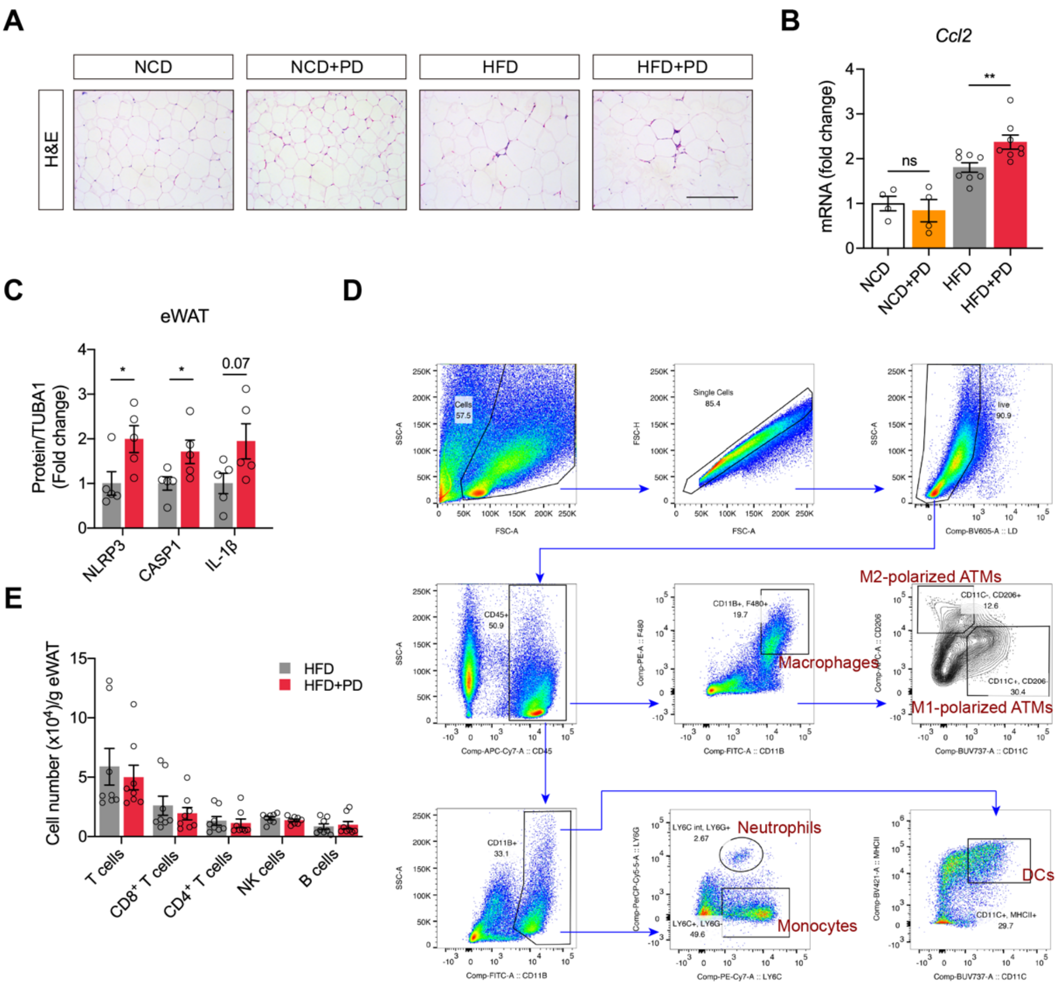

Figure S6. Analysis of immune cell infiltration in adipose tissue. (A) Representative H&E staining of sWAT sections. Scale bar, 200 µm. (B) qRT-PCR analysis of *Ccl2*, a chemokine that regulates the migration and infiltration of macrophages, in sWAT. (C) Densitometric quantification of the immunoblots exemplified in Figure 3D. (D) Flow cytometry gating strategy for myeloid cells in eWAT. After excluding debris, doublets, and dead cells, $CD45^+$ cells were selected. Adipose tissue macrophages (ATMs) were gated as $CD11b^+F4/80^+$ cells and further divided into M1 and M2 subsets based on CD11c and CD206 expression. Among the $CD45^+CD11b^+$ cells, DCs ($MHCII^+CD11c^+$), neutrophils ($Ly6G^+$), and monocytes ($Ly6G^-Ly6C^+$) were subclassified. (E) Quantification of the numbers of the indicated lymphocyte populations in eWAT. Data are presented as mean ± SEM. Each data

point represents an individual mouse. Unpaired Student's *t*-test was used for statistical analysis. ns, not significant. $^{*}p < 0.05$, $^{**}p < 0.01$.

Figure S7

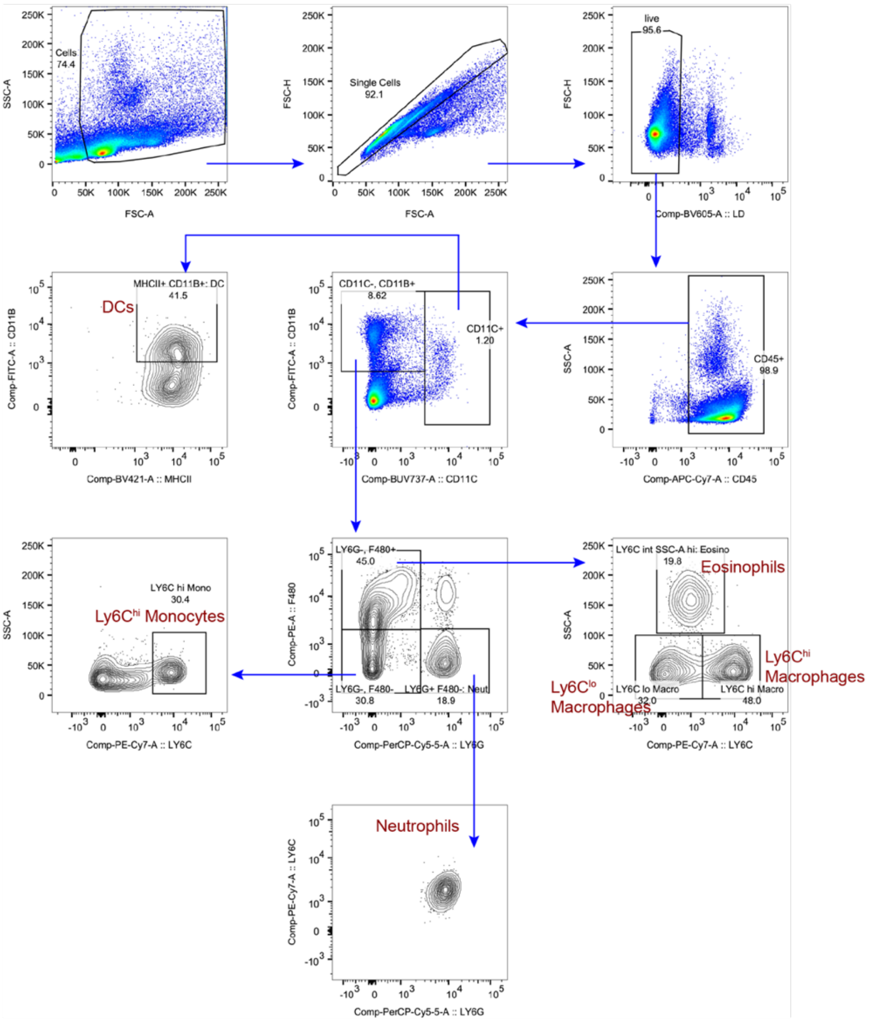

Figure S7. Flow cytometry gating strategy for myeloid cells in the spleen. CD45^{+} cells were selected after excluding debris, doublets, and dead cells. Within the CD11c^{+} population, DCs were identified as CD11b^{+}MHCII^{+}. Among CD11c^{-}CD11b^{+} cells, neutrophils were defined as Ly6G^{+}F4/80^{-} (Ly6C^{int}). The Ly6G^{-}F4/80^{+} population was subdivided into three subsets based on Ly6C and SSC: eosinophils (Ly6C^{int}SSC^{hi}), Ly6C^{hi} macrophages (Ly6C^{hi}SSC^{lo}), and Ly6C^{lo} macrophages (Ly6C^{lo}SSC^{lo}). Ly6C^{hi} monocytes were identified within the Ly6G^{-}F4/80^{-} population as Ly6C^{hi}SSC^{lo}.

Figure S8

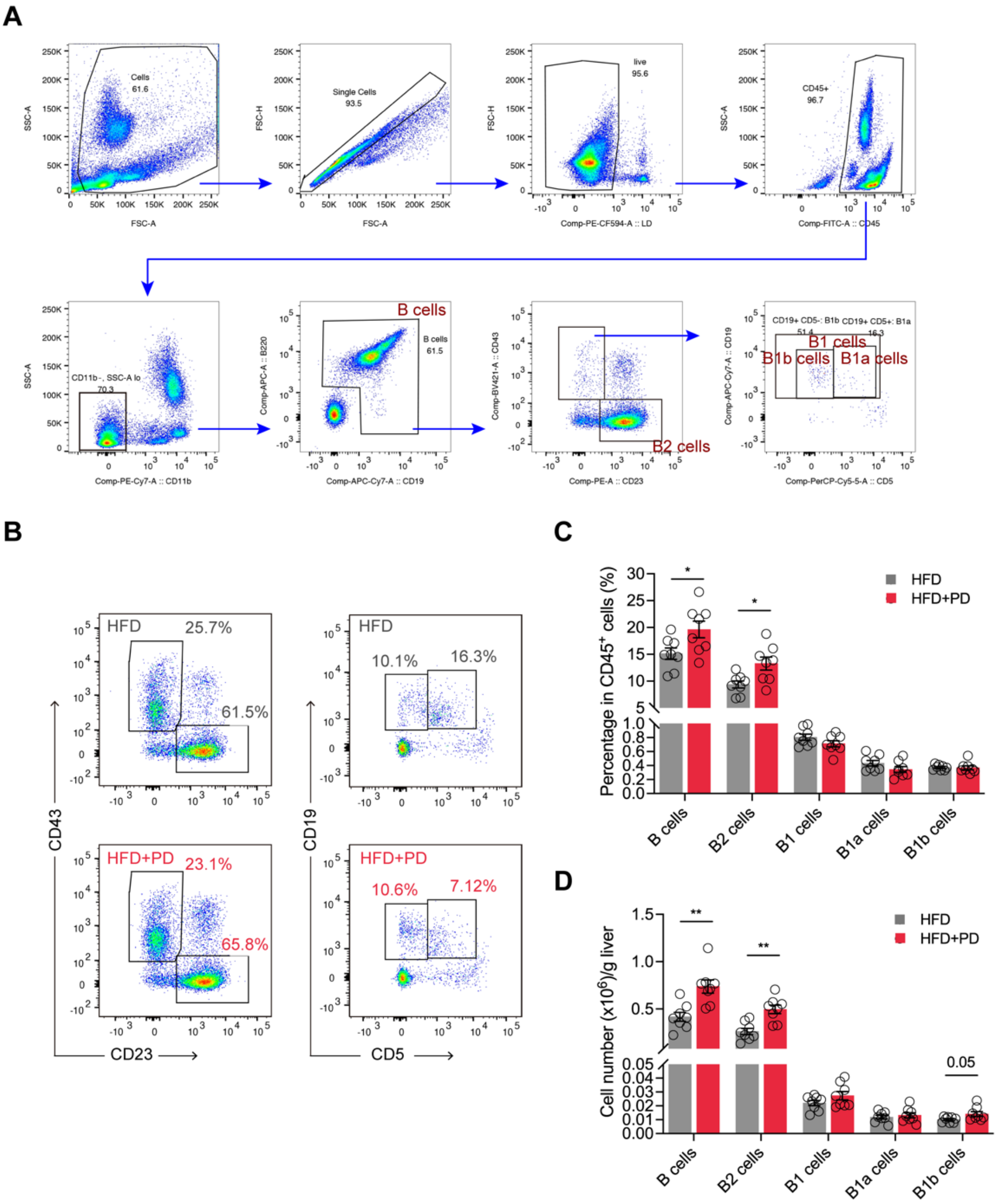

Figure S8. Flow cytometric analysis for B cell subsets in the periphery. (A) Gating strategy for B1 and B2 cells in the peripheral blood and liver. After gating for singlets, live cells, and CD45$^+$ cells, B cells were separated from CD11b$^-$ populations by gating on CD19$^+$/B220$^+$ cells. B2 cells were gated as CD23$^+$CD43$^-$ cells. B1 cells were gated as CD23$^-$CD43$^+$CD19$^+$ cells, which were further divided into B1a and B1b subsets based on CD5 expression. (B) Representative flow cytometry plots showing the proportions of B2, B1a, and B1b cells among B cells in the liver. (C) Quantification of B cells and their main subsets as percentages of CD45$^+$ cells in the liver. (D)

Quantification of the numbers of B cells and their main subsets in the liver. Data are presented as mean ± SEM. Each data point represents an individual mouse. Unpaired Student's *t*-test was used for statistical analysis. $^{*}p < 0.05$, $^{**}p < 0.01$.

Figure S9

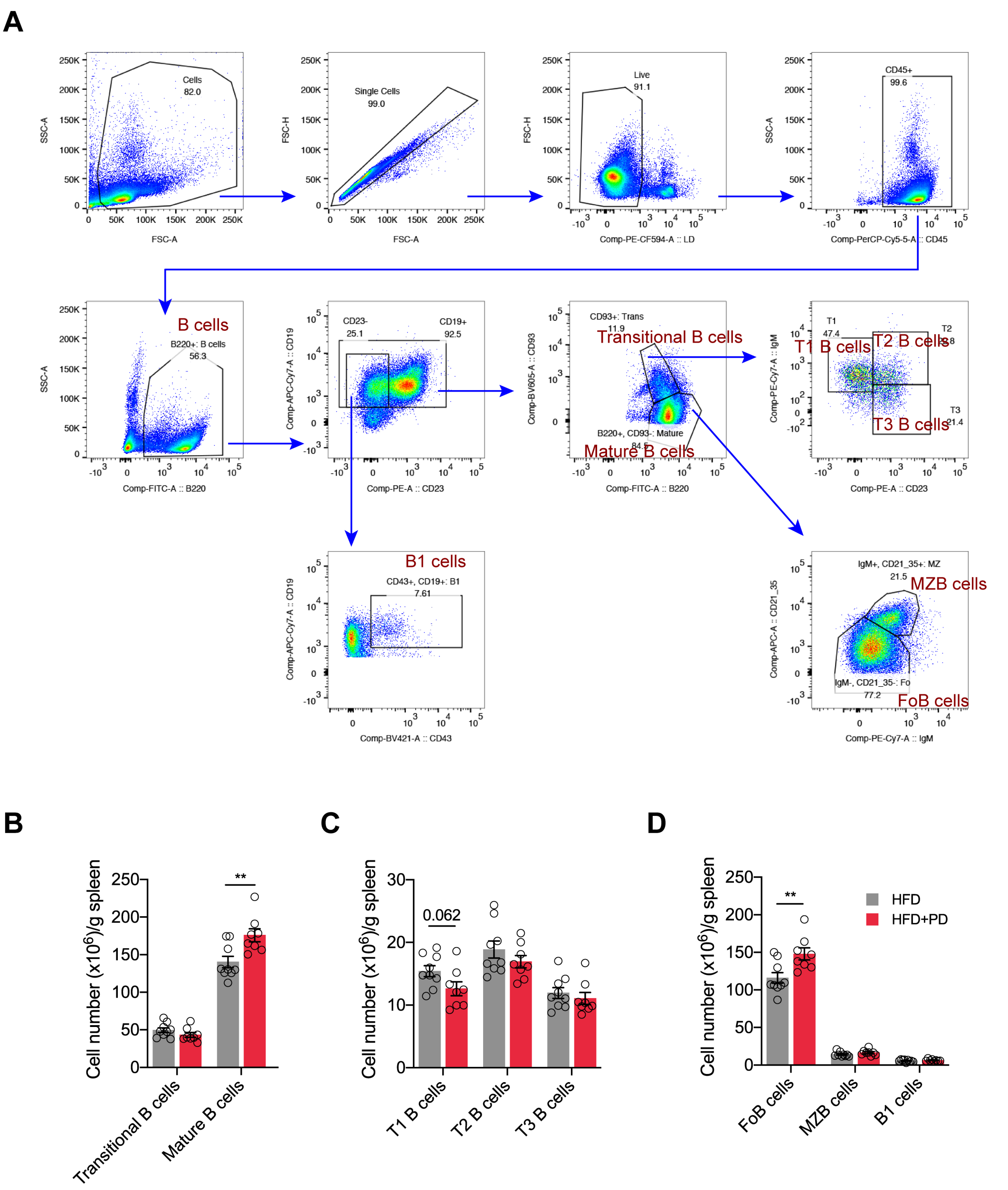

Figure S9. Flow cytometric analysis for B cell maturation in the spleen. (A) Gating strategy for the characterization of splenic B cell maturation. After gating for singlets, live cells, and $CD45^+$ cells, B cells were separated from non–B cells by gating on $B220^+$ cells. B1 cells were identified as $CD23^-CD19^+CD43^+$ cells. To classify B2 populations, $CD19^+$ cells were stratified into transitional ($CD93^+B220^+$) and mature ($CD93^-B220^+$) B cells. Transitional B cells were further subdivided into T1 (IgM^+CD23^-), T2 (IgM^+CD23^+), and T3 ($IgM^{int}CD23^+$) populations. Mature B

cells were further categorized into MZB (CD21/35^{+}IgM^{+}) and FoB (CD21/35^{int}IgM$^{int/+}$) cells. (B–D) Quantification of the numbers of transitional and mature B cells (B), transitional B cell subsets (C), and mature B cell subsets (D) in the spleen. Data are presented as mean ± SEM. Each data point represents an individual mouse. Unpaired Student's *t*-test was used for statistical analysis. $^{**}p < 0.01$.

Figure S10

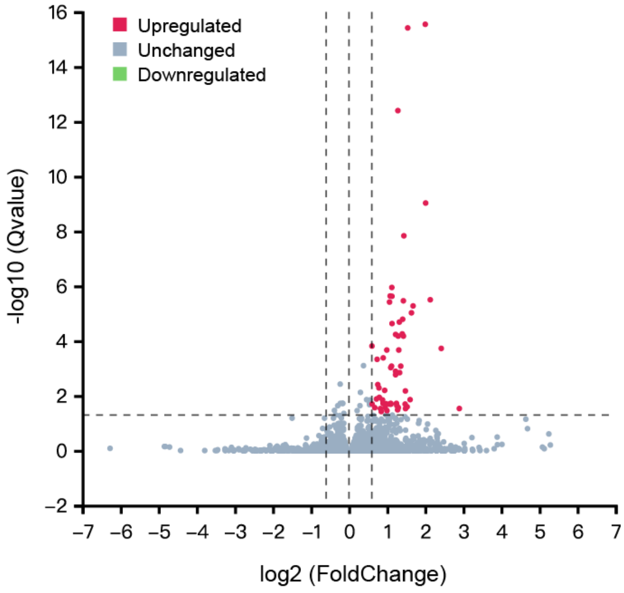

Figure S10. Differential gene expression analysis of splenic B cells from HFD+PD vs. HFD mice. Volcano plot showing 60 significantly upregulated genes (fold change > 1.5) in the HFD+PD group compared with HFD controls.

Table S1. Primer sequences for qRT-PCR.

Gene	Forward sequence (5′–3′)	Reverse sequence (5′–3′)
Il1b	AGCTTCCTTGTGCAAGTGTCT	GACAGCCCAGGTCAAAGGTT
Tlr2	GCTGGAGGACTCCTAGGCT	GTCAGAAGGAAACAGTCCGC
Tlr4	GCTTTCACCTCTGCCTTCAC	GAAACTGCCATGTTTGAGCA
Tlr9	ATGGTTCTCCGTCGAAGGACT	GAGGCTTCAGCTCACAGGG
Il18	TCAAAGTGCCAGTGAACCCC	GGTCACAGCCAGTCCTCTTAC
Il6	GCTACCAAACTGGATATAATCAGGA	CCAGGTAGCTATGGTACTCCAGAA
Tnfa	TCTGTCTACTGAACTTCGGGGTG	ACTTGGTGGTTTGCTACGACG
Nlrp1a	AGGCTCTTTACCCTCTTCTA	ATGTGCTTCTTCTTCTGGTA
Nlrp3	TCCTGCAGAGCCTACAGTTG	ACGCCTACCAGGAAATCTCG
Casp1	CGCCCTGTTGGAAAGGAACT	CCCTCAGGATCTTGTCAGCC
Casp11	CCCAAAGTCATCATTGTGC	CCTCCTGTTTTGTCTCGGTA
Il18r1	GTGCACAGGAATGAAACAGC	ATTTAAGGTCCAATTGCGACGA
Il18rap	AGACTACTTCCTGAGCACAAGA	TGTCCTTACCAATGGTTCTCACT
Srebp1	GAGCCATGGATTGCACATTT	CTCAGGAGAGTTGGCACCTG
Fasn	CGGAAACTTCAGGAAATGTCC	TCAGAGACGTGTCACTCCTGG
Acaca	TGAGGAGGACCGCATTTATC	CATGGGATGGCAGTAAGGTC
Ccl2	CATCCACGTGTTGGCTCA	GATCATCTTGCTGGTGAATGA
Gadph	AGGTCGGTGTGAACGGATTTG	GGGGTCGTTGATGGCAACA
